# The non-invasive evaluation technique of patellofemoral joint stress: a systematic literature review

**DOI:** 10.3389/fbioe.2023.1197014

**Published:** 2023-06-29

**Authors:** Baofeng Wang, Zheng Mao, Jiaming Guo, Jin Yang, Shengnian Zhang

**Affiliations:** Key Laboratory of Exercise and Health Sciences of Ministry of Education, Shanghai University of Sport, Shanghai, China

**Keywords:** patellofemoral joint stress, analytical model, musculoskeletal model, discrete element analysis, finite element analysis

## Abstract

**Introduction:** Patellofemoral joint stress (PFJS) is an important parameter for understanding the mechanism of patellofemoral joint pain, preventing patellofemoral joint injury, and evaluating the therapeutic efficacy of PFP rehabilitation programs. The purpose of this systematic review was to identify and categorize the non-invasive technique to evaluate the PFJS.

**Methods:** Literature searches were conducted from January 2000 to October 2022 in electronic databases, namely, PubMed, Web of Science, and EBSCO (Medline, SPORTDiscus). This review includes studies that evaluated the patellofemoral joint reaction force (PJRF) or PFJS, with participants including both healthy individuals and those with patellofemoral joint pain, as well as cadavers with no organic changes. The study design includes cross-sectional studies, case-control studies, and randomized controlled trials. The JBI quality appraisal criteria tool was used to assess the risk of bias in the included studies.

**Results:** In total, 5016 articles were identified in the database research and the citation network, and 69 studies were included in the review.

**Discussion:** Researchers are still working to improve the accuracy of evaluation for PFJS by using a personalized model and optimizing quadriceps muscle strength calculations. In theory, the evaluation method of combining advanced computational and biplane fluoroscopy techniques has high accuracy in evaluating PFJS. The method should be further developed to establish the “gold standard” for PFJS evaluation. In practical applications, selecting appropriate methods and approaches based on theoretical considerations and ecological validity is essential.

## 1 Introduction

Patellofemoral pain (PFP), a frequent complaint in orthopedic practice, is associated with 25%–40% of knee injuries (Liao et al., 2015). This pain is aggravated by various activities that load the joint (e.g., squatting, running, ascending/descending stairs), greatly limiting the daily activities of individuals with PFP (Fick et al., 2022). There is no consensus on the exact mechanism of PFP development (Vannatta and Kernozek, 2015). However, a prevailing theory holds that PFP develops in response to increased patellofemoral joint stress (PFJS) (Salsich and Perman, 2007). Chronic overuse of the patellofemoral joint has been related to increased intraosseous pressures and pain, microfractures, increased bone metabolism, and increased bone water content, all of which have pathologically detrimental effects on the subchondral bone (Ho et al., 2014). Studies have reported that individuals with PFP exhibit elevated PFJS during walking and running when compared with those in pain-free individuals (Farrokhi et al., 2011b; Liao et al., 2015). Based on the associations, PFJS seems to be an important factor in assessing the load on the patellofemoral joint. It can be helpful in preventing patellofemoral joint injuries, evaluating the effectiveness of PFP rehabilitation programs.

The analytical model is currently the most widely used method for evaluating the PFJS, which is based on the formula obtained from previous cadaver experiments. The classic analytical model has several significant limitations, including the failure to account for the synergistic contraction of knee joint muscles and the consideration of only sagittal plane factors in relation to PFJS (Bonacci et al., 2014; Atkins et al., 2019). To achieve greater accuracy in assessing PFJS, various methods have been developed, including musculoskeletal models, discrete element analysis (DEA), and finite element analysis (FEA). Nunes et al. systematically reviewed the literature which was utilized analytical models to evaluate PFJS and described the possible best paradigm to evaluate PFJS (Nunes et al., 2018). An important limitation of the review is its relatively narrow definition of the methods of PFJS. Nunes et al. do not take into consideration the methods of musculoskeletal modeling, DEA, and FEA. It is necessary to carry out a new literature review due to the progress of PFJS evaluation technology and the increase in the number of studies since the publication of the previous review.

The primary goal of the present systematic review was to identify and categorize the methods developed and used to evaluate PFJS comprehensively by taking into consideration all important aspects of PFJS (e.g., synergistic contraction, variables of the coronal plane, and 3D geometry of bones). This paper will help researchers fully understand the assessment schemes for patellofemoral joint stress and different optimization strategies, while also proposing future research directions.

## 2 Methods

While conducting this review, we followed the recommendations of the PRISMA statement (Supplementary Table S6) (Liberati et al., 2009).

### 2.1 Search strategy

Literature searches were conducted from January 2000 to October 2022 in electronic databases, namely, PubMed, Web of Science, and EBSCO (Medline, SPORTDiscus). The following search terms were used: (“patellofemoral” OR “patellar”) AND (“stress” OR “loading” OR “force”). In addition, the reference lists of the included articles were investigated to detect additional relevant articles that could not be found via the initial electronic search strategy.

### 2.2 Inclusion and exclusion criteria

Those studies evaluating the patellofemoral joint reaction force (PJRF) or PFJS were included, whose participants were healthy individuals, individuals with patellofemoral joint pain, and cadavers (no organic change) (Table 1). Studies published in any language other than English, review papers, book chapters, conference abstracts, commentaries, and study protocols were excluded.

**TABLE 1:** Characteristics of the literature search.

Keywords for literature search	(“Patellofemoral” OR “patellar”) AND (“stress” OR “loading” OR “force”)
Databases	PubMed, EBSCO, Web of Science
Language	English only
Document type	Peer-reviewed empirical article
Inclusion criteria	Population: Healthy and patellofemoral pain adults; cadavers
	Intervention: not necessary
	Comparison: not necessary
	Outcome: objective evaluation of patellofemoral joint reaction force or stress
Exclusion criteria	Dissertations, theoretical papers, conference materials, non-English articles

Two researchers evaluated the search results independently, resolving any differences through consultation. The first step involved eliminating duplicate articles, which was carried out using EndNote and focused on the titles and abstracts. Next, the researchers conducted an initial screening of the titles and removed irrelevant articles. They then read the abstracts and full texts of relevant articles to screen for inclusion based on predefined criteria. Finally, the researchers confirmed the articles that met the criteria and made the final decision on inclusion.

### 2.3 Evaluation of the methodological quality

The included studies were subjected to quality assessment based on the JBI quality appraisal criteria (Barker et al., 2023). The assessment criteria comprised 8–13 items, including the source and characteristics of the study population, control of confounding factors, measurement of outcome indicators, and other relevant aspects. Evaluators provided judgments of “yes”, “no”, “unclear”, or “not applicable” for each item. To evaluate the quality of the included studies, two independent reviewers conducted assessments, and a third reviewer was consulted when there was disagreement.

### 2.4 Data extraction

The basic information (i.e., population, gender, age, height, weight) of participants, PJRF and PFJS calculation methods (quadriceps muscle strength, quadriceps muscle effective lever arm, musculoskeletal model, cartilage mechanics, etc.) and primary outcomes (peak knee flexion moment, peak PJRF, peak PFJS, etc.) were extracted. For studies of setting up training or additional equipment interventions, only the PJRF or PFJS before training or without equipment factors were extracted. Where necessary, WebPlotDigitizer v4.5 was used to extract means and standard deviation from figures in the manuscripts (Whitehead et al., 2018).

## 3 Results

### 3.1 Included studies

**FIGURE 1:**
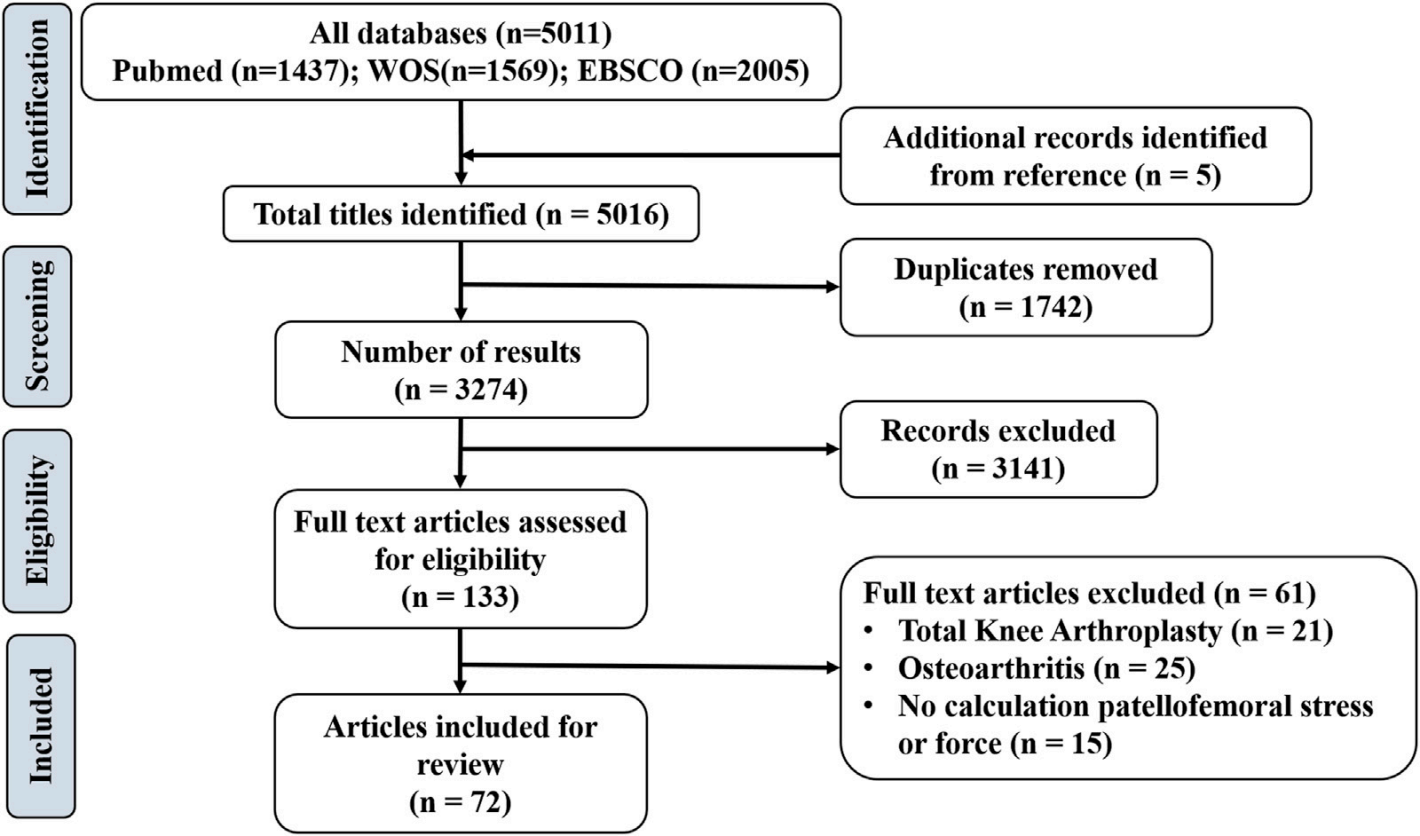
Flow chart of literature search.

A total of 5,016 articles were identified overall based on database search and citation network analysis. After removing the duplicates, 3,274 remained. After reading the titles/abstracts, a further 3,141 articles were excluded because they did not meet the inclusion criteria. Based on the full texts of the remaining 133 articles, 61 studies were excluded. In total, 72 studies were included in the review (Figure 1). Among them, 48 studies evaluated PFJS by analytical model; eight studies evaluated PJRF or PFJS by establishing a musculoskeletal model; four studies evaluated PJRF or PFJS by DEA; 12 studies evaluated PJRF or PFJS by FEA.

### 3.2 Study characteristics

Regarding the methodological quality of the included studies, the majority of studies scored well in terms of the source and characteristics of the study population, measurement of outcome indicators, and other relevant aspects. However, there was a significant deficiency in considering and controlling for confounding factors (Tables 2–4). Among the 72 included studies, 1,432 participants (453 males and 979 females) and 27 cadaveric knees were evaluated regarding PFJS. Two studies assessed PFJF or PFJS in older adults (Hu et al., 2018; Gustafson et al., 2021) (67.7–70 years) and 66 studies assessed PFJF or PFJS calculations in younger adults (18.3–37.1 years). Among the 69 included studies, the health condition of the participants included two categories: 1) individuals with PFP (*n* = 204); 2) individuals without knee injury (*n* = 1,228). The activity condition of the population includes: 1) active population (*n* = 113); 2) high-level athletes (*n* = 88); 3) recreational runners (*n* = 498); 4) no mention of physical activity (*n* = 703) (Supplementary Table S1).

**TABLE 2:** Methodological quality of cross-sectional studies.

	Were the criteria for inclusion in the sample clearly defined?	Were the study subjects and the setting described in detail?	Was the exposure measured in a valid and reliable way?	Were objective, standard criteria used for measurement of the condition?	Were confounding factors identified?	Were strategies to deal with confounding factors stated?	Were the outcomes measured in a valid and reliable way?	Was appropriate statistical analysis used?
Starbuck (2021)	Y	N	Y	Y	N	N	Y	Y
Zavala (2021)	Y	N	Y	Y	N	N	Y	Y
Thomeer (2020)	Y	N	Y	-	N	N	Y	Y
Almonroeder (2020)	Y	N	Y	Y	N	N	Y	Y
Goulette (2021)	Y	N	Y	Y	N	N	Y	Y
Kujawa (2020)	Y	U	Y	Y	N	N	Y	Y
Ristow (2020)	Y	Y	Y	Y	N	N	Y	Y
Atkins (2019)	Y	N	Y	Y	N	N	Y	Y
Dos Santos (2019)	Y	U	Y	Y	Y	N	Y	Y
Ho (2018)	Y	Y	Y	Y	Y	N	Y	Y
Bonacci (2018)	Y	Y	Y	Y	Y	Y	Y	Y
Boyer (2018)	Y	U	Y	Y	N	N	Y	Y
Kernozek (2018)	Y	U	Y	Y	N	N	Y	Y
van Rossom (2018)	Y	U	Y	Y	N	N	Y	Y
Hu (2018)	Y	Y	Y	Y	N	N	U	Y
Liao (2018b)	Y	U	Y	Y	N	N	Y	Y
Esculier (2017)	Y	U	Y	Y	N	N	Y	Y
Hofmann (2017)	Y	U	Y	Y	N	N	Y	Y
Almonroeder (2017)	Y	U	Y	Y	N	N	Y	Y
Sinclair (2016a)	Y	U	Y	Y	N	N	Y	Y
Willy (2016)	Y	Y	Y	Y	N	N	Y	Y
Alexander and Schwameder (2016)	Y	U	Y	Y	N	N	Y	Y
Sinclair and Selfe (2015)	Y	U	Y	Y	N	N	Y	Y
Sinclair (2015)	Y	U	Y	Y	N	N	Y	Y
Willson (2015a)	Y	U	Y	Y	N	N	Y	Y
Willson (2015b)	Y	U	Y	Y	N	N	Y	Y
Kernozek (2015)	Y	U	Y	Y	N	N	Y	Y
Vannatta (2015)	Y	U	Y	Y	N	N	Y	Y
Besier (2015)	Y	U	Y	Y	N	N	Y	Y
Lenhart (2015a)	Y	N	Y	Y	N	N	Y	Y
Lenhart (2015b)	Y	U	Y	Y	N	N	Y	Y
Liao (2015)	Y	Y	Y	Y	N	N	Y	Y
Shah (2015)	N	N	Y	Y	N	N	Y	Y
Islam (2015)	Y	U	Y	Y	N	N	Y	Y
Willson (2014)	Y	U	Y	Y	N	N	Y	Y
Lenhart (2014)	Y	U	Y	Y	N	N	Y	Y
Powers (2014)	Y	U	Y	Y	N	N	Y	Y
Teng (2014)	Y	U	Y	Y	N	N	Y	Y
Chen (2014)	Y	U	Y	Y	N	N	Y	Y
Sinclair (2014)	Y	U	Y	Y	N	N	Y	Y
Bonacci (2014)	Y	U	Y	Y	Y	N	Y	Y
Kulmala (2013)	Y	U	Y	Y	Y	N	N	Y
Elias (2013)	U	U	Y	Y	Y	N	N	Y
Ho (2012)	Y	U	Y	Y	Y	N	N	Y
Chinkulprasert (2011)	Y	U	Y	Y	Y	N	N	Y
Elias (2010)	U	U	Y	Y	Y	N	N	Y
Whyte (2010)	Y	U	Y	Y	Y	N	N	Y
Escamilla (2009)	Y	U	Y	Y	Y	N	N	Y
Escamilla (2008b)	Y	U	Y	Y	Y	N	N	Y
Escamilla (2008a)	Y	U	Y	Y	Y	N	N	Y
Besier (2008)	Y	U	Y	Y	Y	N	N	Y
Fernandez (2008)	Y	U	Y	Y	Y	N	N	Y
Besier (2005)	U	U	Y	Y	Y	N	N	Y
Powers (2004a)	Y	U	Y	Y	Y	N	N	Y
Power (2004b)	Y	U	Y	Y	Y	N	N	Y
Elias (2004)	Y	U	Y	Y	Y	N	N	Y
Wallace (2002)	Y	U	Y	Y	Y	N	N	Y
Salem (2001)	Y	U	Y	Y	Y	N	N	Y

Note: Y: yes, clear report; N: no, not reported; U, unclear; “-”, not applicable

**TABLE 3:** Methodological quality of case-control studies.

	Were the groups comparable other than the presence of disease in cases or the absence of disease in controls?	Were cases and controls matched appropriately?	Were the same criteria used for identification of cases and controls?	Was exposure measured in a standard, valid and reliable way?	Was exposure measured in the same way for cases and controls?	Were confounding factors identified?	Were strategies to deal with confounding factors stated?	Were outcomes assessed in a standard, valid and reliable way for cases and controls?	Was the exposure period of interest long enough to be meaningful?	Was appropriate statistical analysis used?
Gustafson (2021)	Y	Y	Y	Y	Y	N	N	Y	U	Y
Liao (2019)	Y	Y	Y	Y	Y	N	N	Y	U	Y
Pal (2019)	Y	Y	Y	Y	Y	Y	N	Y	U	Y
Waiteman (2018)	Y	Y	Y	Y	Y	N	N	Y	U	Y
Teng (2018)	N	U	Y	Y	Y	Y	N	Y	U	Y
Liao (2018a)	Y	Y	Y	Y	Y	Y	N	Y	Y	Y
Wirtz (2012)	Y	Y	Y	Y	Y	Y	N	Y	Y	Y
Farrokhi (2011b)	Y	Y	Y	Y	Y	Y	N	Y	Y	Y
Brechter (2002a)	Y	Y	Y	Y	Y	Y	N	Y	Y	Y
Brechter (2002b)	Y	Y	Y	Y	Y	Y	N	Y	Y	Y

Note: Y: yes, clear report; N: no, not reported; U, unclear; “-”, not applicable

**TABLE 4:** Methodological quality of randomized controlled trials.

	Was true randomization used for assignment of participants to treatment groups?	Was allocation to treatment groups concealed?	Were treatment groups similar at the baseline?	Were participants blind to treatment assignment?	Were those delivering the treatment blind to treatment assignment?	Were treatment groups treated identically other than the intervention of interest?	Were outcome assessors blind to treatment assignment?	Were outcomes measured in the same way for treatment groups?	Were outcomes measured in a reliable way	Was follow up complete and if not, were differences between groups in terms of their follow up adequately described and analysed?	Were participants analysed in the groups to which they were randomized?	Was appropriate statistical analysis used?	Was the trial design appropriate and any deviations from the standard RCT design (individual randomization, parallel groups) accounted for in the conduct and analysis of the trial?
Wang (2020)	Y	Y	Y	Y	N	N	N	Y	Y	N	Y	Y	Y
Sinclair (2018)	N	Y	Y	N	N	Y	U	Y	Y	U	Y	Y	Y
Sinclair (2016b)	N	N	Y	N	N	Y	N	Y	Y	U	Y	Y	Y
Peng (2015)	N	N	Y	N	N	Y	N	Y	Y	U	Y	Y	Y

Note: Y: yes, clear report; N: no, not reported; U, unclear; “-”, not applicable.

### 3.3 Analytical model

The analytical model is based on functional relationships obtained from previous cadaver studies to evaluate PFJS. The computational process of the mathematical model is straightforward, and the input variables (knee flexion angle and knee extension moment) are easily obtainable. Twenty-four studies evaluated the PFJS of running, walking, squatting, lunging, ascending, descending, landing, and ballet dance activities by the analytical model (Table 5). The analytical model process includes:

1) The effective lever arm (L_eff_) of the quadriceps muscle is calculated using nonlinear formulas (formulas 1, 2) based on the degree of knee flexion angle (x).

$$\begin{array}{c} L_{eff}=\left\{\begin{array}{c} 0.036x+3.0\left(0{}^{\circ}\leq x<30{}^{\circ}\right)\\ -0.043x+5.4\left(30{}^{\circ}\leq x<60{}^{\circ}\right)\\ -0.027x+4.3\left(60{}^{\circ}\leq 90{}^{\circ}\right)\\ 2.0\left(90{}^{\circ}\leq x\right) \end{array}\right. \end{array}$$ (1)

$$\begin{array}{c} L_{eff}{=8.0e}^{-5}x^{3}{-0.013x}^{2}+0.28x+0.046 \end{array}$$ (2)

2) quadriceps muscle force (F_Q_) is calculated by dividing the knee extension moment (M_EXT_) by the effective moment arm (formulas 3, 4).

$$\begin{array}{c} F_{Q}=\frac{M_{EXT}}{L_{eff}} \end{array}$$ (3)

$$\begin{array}{c} F_{Q}=\frac{Quadriceps\;force+Hamstring\;force+Gastrocenemius\;force}{L_{eff}} \end{array}$$ (4)

3) the coefficient k of defining the relation between quadriceps force and PJRF is calculated based on the formulas of knee flexion angle (formulas 5formulas –formulas 7).

$$\begin{array}{c} k=\frac{{4.62e}^{-1}{+1.47e}^{-3}{x-3.84e}^{-5}x^{2}}{{1-1.62e}^{-2}x+1.55e^{-4}x^{2}-6.98e^{-7}x^{3}} \end{array}$$ (5)

$$\begin{array}{c} k=\frac{-3.8e^{-5}x^{2}+1.5e^{-3}x+0.462}{-7.0e^{-7}x^{3}+1.6e^{-4}x^{2}+0.016x+1} \end{array}$$ (6)

$$\begin{array}{c} k=2e^{-07}x^{3}-{0.0001x}^{2}+0.0002x+1.15 \end{array}$$ (7)

4) the PJRF is obtained by multiplying the quadriceps muscle force by the coefficient k or by a function of the knee joint flexion angle (formulas 8, 9).

$$\begin{array}{c} {PFJF=2F}_{Q}\sin\left(\frac{30.46+0.53x}{2}\right) \end{array}$$ (8)

$$\begin{array}{c} {PFJF=F}_{Q}•k \end{array}$$ (9)

5) the patellofemoral joint contact area (CA) is calculated based on the formula of knee flexion angle (e10formulas –formulas 18).

$$\begin{array}{c} {CA=0.0781x}^{2}+0.06763x+151.7 \end{array}$$ (10)

$$\begin{array}{c} CA={0.0781x}^{2}+0.6763x+151.7 \end{array}$$ (11)

$$\begin{array}{c} {CA=2.0e}^{-5}x^{4}{-0.0033x}^{3}{+0.1099x}^{2}+3.5273x+81.058 \end{array}$$ (12)

$$\begin{array}{c} {CA=-0.028x}^{2}+4.17x+70.81 \end{array}$$ (13)

$$\begin{array}{c} {CA=-0.258x}^{2}+7.4276x+304.0342 \end{array}$$ (14)

$$\begin{array}{c} {CA=-0.0129x}^{2}+6.4114x+184.9724 \end{array}$$ (15)

$$\begin{array}{c} CA=3.55x+135 \end{array}$$ (16)

$$\begin{array}{c} {CA=-0.0242x}^{2}+7.3142x+303.57 \end{array}$$ (17)

$$\begin{array}{c} CA={0.0157x}^{2}+4.7478x+182.95 \end{array}$$ (18)

6) the PFJS is obtained by dividing the PJRF by the patellofemoral joint contact area (formula 19).

$$\begin{array}{c} PFJS=\frac{PJRF}{CA} \end{array}$$ (19)

**TABLE 5:** Complete methods to calculate patellofemoral joint stress by analytical model.

Literature	The input data	L_eff_	F_Q_	k coefficient	PFJF	Contact area	PFJS
Almonroeder (2020)	Kinematic and kinetic data	Formula 2	Formula 3	Formula 6	Formula 9	Formula 13	Formula 5
Wang (2020)	Kinematic and kinetic data	Formula 1	Formula 3	-	Formula 8	Formula 10	Formula 5
Atkins (2019)	Kinematic and kinetic data	U/C	U/C	U/C	U/C	U/C	Formula 5
Dos Santos (2019)	Kinematic and kinetic data	Formula 2	Formula 3	Formula 5	Formula 9	Formula 11	Formula 5
Waiteman (2018)	Kinematic and kinetic data	U/C	U/C	U/C	U/C	U/C	Formula 5
Bonacci (2018)	Kinematic and kinetic data	U/C	U/C	U/C	U/C	U/C	Formula 5
Ho (2018)	Kinematic and kinetic data	U/C	U/C	U/C	U/C	U/C	Formula 5
Teng (2018)	Kinematic and kinetic data	U/C	U/C	U/C	U/C	U/C	Formula 5
Esculier (2017)	Kinematic and kinetic data	U/C	U/C	U/C	U/C	U/C	Formula 5
Almonroeder (2017)	Kinematic and kinetic data	Formula 2	Formula 3	Formula 5	Formula 9	Formula 11	Formula 5
Hofmann (2017)	Kinematic and kinetic data	U/C	U/C	U/C	U/C	U/C	Formula 5
Sinclair (2016a)	Kinematic and kinetic data	Formula 2	Formula 3	Formula 5	Formula 9	Formula 11	Formula 5
Sinclair (2016b)	Kinematic and kinetic data	Formula 2	Formula 3	Formula 5	Formula 9	Formula 11	Formula 5
Peng (2015)	Kinematic and kinetic data	U/C	U/C	U/C	U/C	U/C	Formula 5
Sinclair (2015)	Kinematic and kinetic data	Formula 2	Formula 3	Formula 5	Formula 9	Formula 11	Formula 5
Sinclair Selfe (2015)	Kinematic and kinetic data	Formula 2	Formula 3	Formula 5	Formula 9	Formula 11	Formula 5
Sinclair (2014)	Kinematic and kinetic data	Formula 2	Formula 3	Formula 5	Formula 9	Formula 11	Formula 5
Willson (2014)	Kinematic and kinetic data	U/C	U/C	U/C	U/C	U/C	Formula 5
Bonacci (2014)	Kinematic and kinetic data	Formula 2	Formula 3	Formula 6	Formula 9	Formula 12	Formula 5
Kulmala (2013)	Kinematic and kinetic data	Formula 2	Formula 3	Formula 5	Formula 9	Formula 11	Formula 5
Ho (2012)	Kinematic and kinetic data	U/C	U/C	U/C	U/C	U/C	Formula 5
Wirtz (2012)	Kinematic and kinetic data	U/C	U/C	U/C	U/C	U/C	Formula 5
Wallace (2002)	Kinematic and kinetic data	Formula 2	Formula 3	Formula 6	Formula 9	U/C	Formula 5
Salem (2001)	Kinematic and kinetic data	Formula 2	Formula 3	Formula 6	Formula 9	U/C	Formula 5
Whyte (2010)	Kinematic and kinetic data	Formula 2	Formula 3	Formula 6	Formula 9	MRI	Formula 5
Powers (2004a)	Kinematic and kinetic data	Formula 2	Formula 3	Formula 6	Formula 9	MRI	Formula 5
Power (2004b)	Kinematic and kinetic data	Formula 2	Formula 3	Formula 6	Formula 9	MRI	Formula 5
Brechter (2002a)	Kinematic and kinetic data	Formula 2	Formula 3	Formula 5	Formula 9	MRI	Formula 5
Brechter (2002b)	Kinematic and kinetic data	Formula 2	Formula 3	Formula 5	Formula 9	MRI	Formula 5
Starbuck (2021)	kinematic and kinetic data	Formula 2	Formula 4	Formula 5	Formula 9	Formula 14 and 15	Formula 5
Zavala (2021)	kinematic and kinetic data	U/C	Formula 4	U/C	U/C	U/C	Formula 5
Willson (2015a)	Kinematic and kinetic data	U/C	Formula 4	U/C	U/C	U/C	Formula 5
Willson (2015b)	Kinematic and kinetic data	U/C	Formula 4	U/C	U/C	U/C	Formula 5
Sinclair (2018)	Kinematic and kinetic data	U/C	Formula 4	U/C	U/C	U/C	Formula 5
Willy (2016)	Kinematic and kinetic data	U/C	Formula 4	U/C	U/C	U/C	Formula 5
Teng (2014)	Kinematic, kinetic and EMG data	U/C	SIMM (consider co-contraction)	U/C	U/C	U/C	Formula 5
Powers (2014)	Kinematic, kinetic and EMG data	U/C	SIMM (consider co-contraction)	U/C	U/C	U/C	Formula 5
Chinkulprasert (2011)	Kinematic, kinetic and EMG data	U/C	SIMM (consider co-contraction)	U/C	U/C	U/C	Formula 5
Escamilla (2009)	Kinematic, kinetic and EMG data	U/C	adjusted by EMG–force relationship	U/C	U/C	Formula 16	Formula 5
Escamilla (2008a)	Kinematic, kinetic and EMG data	U/C	adjusted by EMG–force relationship	U/C	U/C	Formula 16	Formula 5
Escamilla (2008b)	Kinematic, kinetic and EMG data	U/C	adjusted by EMG–force relationship	U/C	U/C	Formula 16	Formula 5
Goulette (2021)	Kinematic and kinetic data	-	static optimisation	Formula 5	Formula 9	Formula 11	Formula 5
Kujawa (2020)	Kinematic and kinetic data	-	static optimisation	Formula 5	Formula 9	Formula 11	Formula 5
Ristow (2019)	Kinematic and kinetic data	-	static optimisation	Formula 5	Formula 9	Formula 11	Formula 5
Boyer (2018)	Kinematic and kinetic data	-	static optimisation	Formula 7	Formula 9	Formula 17 and 18	Formula 5
Kernozek (2018)	Kinematic and kinetic data	-	static optimisation	Formula 5	Formula 9	Formula 11	Formula 5
Kernozek (2015)	Kinematic and kinetic data	-	static optimisation	Formula 5	Formula 9	Formula 11	Formula 5
Vannatta (2015)	Kinematic and kinetic data	-	static optimisation	Formula 5	Formula 9	Formula 11	Formula 5

${Formula\;1\;L}_{eff}=\left\{\begin{array}{c} 0.036x+3.0\left(0{}^{\circ}\leq x<30{}^{\circ}\right)\\ -0.043x+5.4\left(30{}^{\circ}\leq x<60{}^{\circ}\right)\\ -0.027x+4.3\left(60{}^{\circ}\leq x<90{}^{\circ}\right)\\ 2.0\left(90{}^{\circ}\leq x\right) \end{array}\right.$

$Formula\;2\;L_{eff}{=8.0e}^{-5}x^{3}{-0.013x}^{2}+0.28x+0.046$

${Formula\;3\;F}_{Q}=M_{EXT}/L_{eff}$

$Formula\;4\;F_{Q}={\left(Quadriceps\;force+Hamstring\;force+Gastrocnemius\;force\right)/L}_{eff}$

$Formula\;5\;k={\left(4.62e\right.}^{-1}{+1.47e}^{-3}{x-3.84e}^{-5}x^{2})/\left({1-1.62e}^{-2}x+1.55e^{-4}x^{2}-6.98e^{-7}x^{3}\right)$

$\left.Formula\;6\;{k=(-3.8e}^{-5}x^{2}{+1.5e}^{-3}{x+0.462)/(-7.0e}^{-7}x^{3}{+1.6e}^{-4}x^{2}+0.016x+1\right)$

$Formula\;7\;k=2e^{-07}x^{3}--{0.0001x}^{2}+0.0002x+1.15$

$Formula\;8\;{PFJF=2F}_{Q}\sin\left(\frac{30.46+0.53x}{2}\right)$

$Formula\;9\;{PFJF=F}_{Q}•k$

$Formula\;10\;{CA=0.0781x}^{2}+0.06763x+151.75$

$Formula\;11\;CA={0.0781x}^{2}+0.6763x+151.75$

$Formula\;12\;{CA=2.0e}^{-5}x^{4}{-0.0033x}^{3}{+0.1099x}^{2}+3.5273x+81.058$

$Formula\;13\;{CA=-0.028x}^{2}+4.17x+70.81$

$Formula\;14\;{CA=-0.258x}^{2}+7.4276x+304.0342$

$Formula\;15\;{CA=-0.0129x}^{2}+6.4114x+184.9724$

Formula 16 CA=3.55x+135

$Formula\;17\;{CA=-0.0242x}^{2}+7.3142x+303.57$

$Formula\;18\;CA={0.0157x}^{2}+4.7478x+182.95$

Formula 19 PFJS=PJRF/CA

Note: L_eff_, Quadriceps muscle effective lever arm; F_Q_, Quadriceps force; k, k coefficient; x, knee flexion angle; PFJF, Patellofemoral joint force; CA, Contact area; PFJS, Patellofemoral joint stress; EMG, Electromyographic; MRI, Magnetic resonance imaging; U/C, formulas have not been presented but relevant references have been provided; “-“, No calculation required.

Fourteen out of 24 studies evaluated the PFJS of recreational runners during running at speeds ranging from 2.3 to 4 m/s, and the result of PFJS was 6.00–20.6 MPa (Wirtz et al., 2012; Kulmala et al., 2013; Bonacci et al., 2014; Sinclair, 2014; Willson et al., 2014; Sinclair and Selfe, 2015; Sinclair J. et al., 2016; Sinclair J. K. et al., 2016; Almonroeder and Benson, 2017; Esculier et al., 2017; Bonacci et al., 2018; Ho et al., 2018; Dos Santos et al., 2019; Wang et al., 2020). One out of 24 studies evaluated the PFJS of healthy females during ascend stair, and the result of PFJS was 6.61–9.99 MPa (Atkins et al., 2019). One out of 24 studies evaluated the PFJS of females with PFP and pain free during descent stair, and the result of PFJS was 9.2–12.5 MPa (Waiteman et al., 2018). Three out of 24 studies evaluated the PFJS of healthy participants at squat, and the result of PFJS was 8.8–12.34 MPa (Salem and Powers, 2001; Wallace et al., 2002; Almonroeder and Benson, 2017). Two out of 24 studies evaluated the PFJS of healthy individuals at walking, and the result of PFJS was 2.6–3.5 MPa (Ho et al., 2012; Teng et al., 2018). Three out of 24 studies evaluated the PFJS at lunges (Hofmann et al., 2017), ballet (Peng et al., 2015) and landings (Sinclair et al., 2015), and the result of PFJS was 7.17–26.71 MPa (Supplementary Table S2).

Five studies evaluated PFJS based on the analytical model but adjusted for patellofemoral contact area using magnetic resonance imaging (MRI) (Table 5). Two of these studies evaluated the PFJS during walking, and the result of PFJS was 2.33–6.61 MPa (Brechter and Powers, 2002b; Powers et al., 2004a). Two of these studies evaluated the PFJS at ascending, and the result of PFJS was 6.46–6.97 MPa (Brechter and Powers, 2002a; Powers et al., 2004b). One of these studies evaluated the PFJS at squat, and the result of PFJS was 23.62 ± 6.89 MPa (Whyte et al., 2010) (Supplementary Table S2).

Twelve studies calculated a quadriceps muscle strength by modified methods, which accounted for co-contraction of the hamstrings and gastrocnemius muscles (Table 5). Six of these studies evaluated the PFJS during running, and the result of PFJS was 5.1–21.5 MPa (Teng and Powers, 2014; Willson et al., 2015a; Willson et al., 2015b; Willy et al., 2016; Sinclair et al., 2018; Starbuck et al., 2021). Three of these studies evaluated the PFJS at squat, and the result of PFJS was 7.09–12.3 MPa (Escamilla et al., 2009; Powers et al., 2014; Zavala et al., 2021). Two of these studies evaluated the PFJS in the lunge, and the result of PFJS was 5.09–5.45 MPa (Escamilla et al., 2008a; Escamilla et al., 2008b). One of these studies evaluated the PFJS at ascending and the result of PFJS was 9.49 MPa (Chinkulprasert et al., 2011) (Supplementary Table S2).

Six studies estimated the muscle strength forces from joint moments by minimizing a static cost function (Kernozek et al., 2015; Vannatta and Kernozek, 2015; Boyer and Derrick, 2018; Kernozek et al., 2018; Kujawa et al., 2020; Goulette et al., 2021) (Table 5). The total quadriceps force was obtained by summing the muscle forces of the rectus femoris, vastus medialis, vastus lateralis, and vastus intermedius. Studies evaluated the PFJS at running (Vannatta and Kernozek, 2015; Boyer and Derrick, 2018; (Kujawa et al., 2020), squatting (Kernozek et al., 2015; Kernozek et al., 2018), lunge (Goulette et al., 2021) activities, respectively, and the result of PFJS was 5.0–20.1 MPa (Supplementary Table S2).

### 3.4 Musculoskeletal model

A musculoskeletal model can provide an accurate estimate and more detailed and valid information on lower limb muscle and joint loads (Steele et al., 2012; Haight et al., 2014). The computed forces are useful for understanding the relative demands on muscles and joints, as well as the potential risk and pathologies for injuries (Erdemir et al., 2007). Eight studies built the musculoskeletal models of the knee to evaluate PJRF. Five of these studies performed PFJF calculations for running (Lenhart et al., 2015b), walking (Lenhart et al., 2015a; Hu et al., 2018; van Rossom et al., 2018; Thomeer et al., 2020), squatting (van Rossom et al., 2018), and lunging (van Rossom et al., 2018) based on elastic foundation models, and the result of PJRF was 0.57–5.59 BW (Supplementary Table S3). The elastic modulus of the cartilage used was 5 MPa (Lenhart et al., 2015a; Lenhart et al., 2015b; Hu et al., 2018; Thomeer et al., 2020), 10 MPa (van Rossom et al., 2018), and the Poisson’s ratio was 0.45 (Lenhart et al., 2015a; Lenhart et al., 2015b; van Rossom et al., 2018; Thomeer et al., 2020), 0.46 (Hu et al., 2018). Hu et al. (2018) set the cartilage thickness to 1 mm, Lenhart et al. (2015a) set it to 3 mm, 3.5 mm (Lenhart et al., 2015b), and van Rossom et al. set it to 4 mm (van Rossom et al., 2018). Thomeer et al. (2020) calculated cartilage thickness directly from the MR images (the shortest distance between the bone-cartilage interface in various regions of the knee joint). Three of these studies performed PFJF calculations for running (Chen and Powers, 2014; Lenhart et al., 2014), walking (Chen and Powers, 2014; Alexander and Schwameder, 2016), stair climbing (Chen and Powers, 2014), and descending (Chen and Powers, 2014) based on multibody dynamics and the result of PJRF is 0.86–6.72 BW. Among them, Seven studies used the concurrent optimization of muscle activations and kinematics algorithm (Chen and Powers, 2014; Lenhart et al., 2014 (Lenhart et al., 2015b; Alexander and Schwameder, 2016; van Rossom et al., 2018); or used a computed muscle control algorithm to determine the muscle excitations needed to produce the computed kinematic trajectories (Lenhart et al., 2015a; Thomeer et al., 2020). One of eight studies did not describe the method of calculating muscle force (Hu et al., 2018) (Table 6).

**TABLE 6:** Complete methods to calculate patellofemoral joint stress by musculoskeletal model.

Literature	Year	The input data	Musculoskeletal model	Muscle force	Cartilage thicknesses	Elastic modulus	Poisson ratio	PFRF
van Rossom (2018)	2018	Kinematic and GRF data	6 DoF for the patellofemoral joints, 44 musculotendon actuators and 14 bundles of nonlinear springs (representing the major knee ligaments and posterior capsule)	optimization of muscle activations and kinematics algorithm	4 mm	10 MPa	0.45	nonlinear elastic foundation formulation based on the penetration depth between overlapping cartilage surface meshes
Hu (2018)	2018	Kinematic and GRF data	5-DOFs in the patellofemoral joint, 55 muscle-tendon units, The ligaments were the ACL, PCL, MCL and LCL	minimizing a cubic polynomial cost function	elastic layer thicknesses: 1 mm	5 MPa	0.46	elastic foundation contact model
Lenhart (2015a)	2015	subject-specific MRI, kinematic, kinetic and EMG data	individual subject models included natural ligament and bone geometries from MRI	computed muscle control algorithm	3 mm	5 MPa	0.45	non-linear elastic foundation formulation
Thomeer (2020)	2020	subject-specific MRI, kinematic (MoBiX, MCS), kinetic and EMG data	participant-specific geometric models (OpenSim 3.3)	dynamic optimization techniques	measured directly on the MRI	5 MPa	0.45	elastic foundation models
Lenhart (2015b)	2015	Kinematic, GRF data	include 6-DOFs in the patellofemoral joint, 44 musculotendon units and 11 ligaments	computed muscle control algorithm	3.5 mm	5 MPa	0.45	nonlinear elastic foundation formulation based on the penetration depth between overlapping cartilage surface meshes
Alexander (2016)	2016	Kinematic and GRF data	standard model (AMMR 1.6.2, MoCapModel)	minimizing a cubic polynomial cost function	U	U	U	multibody dynamics model
Chen (2014)	2014	subject-specific MRI, kinematic, kinetic and EMG data	individual subject models	static optimization routine	U	U	U	multibody dynamics model
Lenhart (2014)	2014	Kinematic and GRF data	included geometric descriptions of the patellar tendon and 92 additional musculotendon units	optimization of muscle activations and kinematics algorithm	U	U	U	multibody dynamics model

Note: U, unable to determine; GRF, ground reaction force; EMG, electromyographic; MRI, magnetic resonance imaging; ACL, anterior cruciate ligament; PCL, posterior cruciate ligament; MCL, medial collateral ligament; LCL, lateral collateral ligament.

### 3.5 Discrete element analysis

DEA is a technique that is often used to evaluate joint stress distribution and determine influencing factors. DEA treat the patellofemoral cartilage as a layer of compressive springs separating rigid bones, with the soft tissue restraints treated as tensile springs. Four studies evaluated the PFJS of knee flexion (Elias et al., 2010; Elias and Saranathan, 2013), squat (Elias et al., 2004; Besier et al., 2005), and walk (Gustafson et al., 2021) activities by DEA (Table 7). For each DEA model, cartilage was assigned isotropic linear-elastic material properties, which were assigned an elastic modulus of 2 or 4 MPa, and a Poisson’s ratio of 0.45. The output variables of the DEA include lateral and medial facets PFJS. The result of lateral facet PFJS was 2.55–6.81 MPa. The result of medial facet PFJS was 2.41–4.68 MPa (Supplementary Table S4).

**TABLE 7:** Complete methods to calculate patellofemoral joint stress by discrete element analysis.

Literature	Year	The input data	Musculoskeletal model	Cartilage surface	Cartilage thicknesses (mm)	Elastic modulus (MPa)	Poisson ratio	PFRF	PFJS
Elias (2013)	2013	subject-specific MRI	The bones were considered to be rigid, while the cartilage and, patellar tendon and muscle were represented with compressive and tensile springs, respectively	10,000	U	4	0.45	discrete element analysis	dividing the force within each spring by the area covered by the spring
Elias (2010)	2011	subject-specific MRI	The bones were considered to be rigid, while the cartilage and, patellar tendon and muscle were represented with compressive and tensile springs, respectively	1,000	-U	4	0.45	discrete element analysis	dividing the force within each spring by the area covered by the spring
Elias (2004)	2004	subject-specific CT	The bones were considered to be rigid, while the cartilage and, patellar tendon and muscle were represented with compressive and tensile springs, respectively	3,000	5	4	0.45	discrete element analysis	dividing the force within each spring by the area covered by the spring
Gustafson (2021)	2021	Kinematics from biplane fluoroscopy, subject-specific CT and MRI	U	U	U	2	0.45	discrete element analysis	dividing the force within each spring by the area covered by the spring

Note: U, unable to determine; CT, computed tomography; MRI, magnetic resonance imaging.

### 3.6 Finite element analysis

FEA are refined computational models that allow the integration of subject-specific musculoskeletal parameters and *in vivo* experimental data, and are of great value for understanding stress distributions in complex biological structures (Fernandez et al., 2008; Farrokhi et al., 2011b). Eleven studies evaluated the PFJS of ascending stair (Fernandez et al., 2008; (Besier et al., 2015; Pal et al., 2019), running (Liao et al., 2018a; Liao and Powers, 2019), and squat (Besier et al., 2008; Fernandez et al., 2008; (Farrokhi et al., 2011b; (Islam et al., 2015 (Liao et al., 2015; Shah et al., 2015); by FEA (Table 8). The cartilage of patella was modeled using homogeneous isotropic tetrahedral or hexahedral continuum elements with an elastic modulus of 4, 5, 7, 12, 25, and 40 MPa and a Poisson ratio of 0.45, 0.46, and 0.47. Concerning the muscle strength calculations, nine studies calculated quadriceps muscle strength by inputting kinematics, kinetics, and EMG data (Liao and Powers, 2019). A study performed FEA simulations by using specific quadriceps muscle strength values (Shah et al., 2015). Another study used a three-dimensional registration technique and linear mapping to investigate the PFJS: the depth of virtual penetration of the patellar cartilage surface into the femoral cartilage surface, which does not require the calculation of muscle strength (Islam et al., 2015) (Supplementary Table S5).

**TABLE 8:** Complete methods to calculate patellofemoral joint stress by finite element analysis.

Literature	Year	The input data	Muscle forces	FE model					PFJF	PFJS
				Volume elements	Edge length (mm)	Connector elements	Elastic modulus (MPa)	Poisson ratio		
Liao (2019)	2019	Segment the geometry of the bones and cartilage of the patellofemoral join; kinematics, kinetics, and EMG data; quadriceps muscle morphology	forward-dynamic equations	tetrahedral	0.75	patellar tendon and quadriceps muscles	4	0.47	Quasi-static, nonlinear finite element solve	
Pal (2019)	2019	Segment the geometry of the bones and cartilage of the patellofemoral join; kinematics, kinetics, and EMG data	EMG-driven musculoskeletal model	eight-noded shell elements	1	patellar tendon and quadriceps muscles	12	0.47	Quasi-static, nonlinear finite element solve	
Liao (2018b)	2018	Segment the geometry of the bones and cartilage of the patellofemoral join; kinematics, kinetics, and EMG data	forward-dynamic equations	tetrahedral	0.75	patellar tendon and quadriceps muscles	25	0.47	Quasi-static, nonlinear finite element solve	
Liao (2018a)	2018	Segment the geometry of the bones and cartilage of the patellofemoral join; kinematics, kinetics, and EMG data; quadriceps muscle morphology	forward-dynamic equations	tetrahedral	0.75	patellar tendon and quadriceps muscles	25	0.47	Quasi-static, nonlinear finite element solve	
Besier (2015)	2015	Segment the geometry of the bones and cartilage of the patellofemoral join; kinematics, kinetics, and EMG data	EMG-driven musculoskeletal model	hexahedral	1	patellar tendon and quadriceps muscles	12	0.47	Quasi-static, finite-sliding simulations	
Liao (2015)	2015	Segment the geometry of the bones and cartilage of the patellofemoral join; kinematics, kinetics, and EMG data; quadriceps muscle morphology	forward-dynamic equations	tetrahedral	0.75	patellar tendon and quadriceps muscles	4	0.47	Quasi-static, nonlinear finite element solve	
Shah (2015)	2015	Segment the geometry of the bones and cartilage of the patellofemoral join and kinematics data	U	hexahedral	0.5	patellar tendon and quadriceps muscles	5	0.45	Quasi-static, nonlinear finite element solve	
Islam (2015)	2015	Segment the geometry of the bones and cartilage of the patellofemoral join and kinematics of joint data	U	tetrahedral	1	U	12	0.45	U	
Farrokhi (2011b)	2011	Segment the geometry of the bones and cartilage of the patellofemoral join; kinematics, kinetics, and EMG data; quadriceps muscle morphology	forward-dynamic equations	tetrahedral	0.75	patellar tendon and quadriceps muscles	4	0.47	Quasi-static, nonlinear finite element solve	
Besier (2008)	2008	Segment the geometry of the bones and cartilage of the patellofemoral join; kinematics, kinetics, and EMG data; quadriceps muscle morphology	forward-dynamic equations	U	U	patellar tendon and quadriceps muscles	7	0.47	Quasi-static, nonlinear finite element solve	
Fernandez (2008)	2008	Segment the geometry of the bones and cartilage of the patellofemoral join; kinematics of jint, kinetics, and EMG data; quadriceps muscle morphology	static optimization	hexahedral	U	patellar tendon and quadriceps muscles	40	0.45	Quasi-static, nonlinear finite element solve	
Besier (2005))	2005	Segment the geometry of the bones and cartilage of the patellofemoral join; kinematics, kinetics, and EMG data; quadriceps muscle morphology	EMG-driven musculoskeletal model	hexahedral	U	patellar tendon and quadriceps muscles	6	0.47	Quasi-static, nonlinear finite element solve	

Note: U, unable to determine; EMG, electromyographic.

## 4 Discussion

Studies of the patellofemoral joint mechanism have particularly concerned the forces and stresses in the joint (van Eijden et al., 1986). Non-invasive evaluation of PFJS will provide important information on the factors contributing to joint load in a special population and may provide data that can be used to guide treatment (Brechter and Powers, 2002b). Thus, it is necessary to evaluate the PFJF or PFJS non-invasively. This review aims to identify and categorize methods used to evaluate PFJS non-invasively, and we found four methodologies: analytical model musculoskeletal model, DEA, and FEA.

At present, there is still no “gold standard” for PFJS assessment because it is impossible to measure the PFJS directly *in vivo*. Accurate assessment of PFJS will aid engineers in the design of better tissue-engineered constructs for cartilage replacement and assist physiotherapists to evaluate the PFP patient’s recovery and design a more effective intervention. Researchers have adopted some techniques to optimize the PFJS assessment program to improve the accuracy of PFJS or expand its scope of application. It is mainly aimed at patella kinematics and quadriceps muscle strength optimization.

The geometry or alignment of the patella affects kinematics, contact mechanics, and strain in the patellar bone, potentially leading to PFP (Brechter and Powers, 2002b; Fitzpatrick and Rullkoetter, 2012). The differences in subject-specific anatomy among the population will cause errors in the process of calculating the PFJS. Researchers use personalized programs to eliminate errors. In the analytical model evaluation scheme, the patellofemoral contact area was estimated using a function of knee flexion angles from 0° to 90°, as previously described in past cadaver studies. It is not clear whether the cadaveric data can represent the characteristics of the patellofemoral joint *in vivo*. Furthermore, in studies of patellofemoral bracing, it is not appropriate to calculate the patellar joint contact area by formula because the patellofemoral bracing will affect the patellar trajectory and change the patellofemoral contact area (Powers et al., 2004a). This implies that the generalized equation for the patellofemoral contact area may not be fully accurate (Zavala et al., 2021). A analytical model based on MRI technology was applied to establish a regression equation of contact area based on their respective sample (Brechter and Powers, 2002a; b; Powers et al., 2004a; Whyte et al., 2010). However, the MRI assessment protocol still has some limitations. For example, participants completed the scan in a relaxed quadriceps state to avoid motion artifacts in the MRI, which may not reflect the patellofemoral joint contact area when the quadriceps is contracted.

The accurate prediction of patellofemoral joint kinematics is a key prerequisite prior to investigating the effects of evaluating the impact of injury and physical activity on the PFJS. The geometry of the patella and femur is an important factor affecting the kinematics of the patella. Yu et al. (2019) study revealed that soft tissue plays a critical role in adjusting patellar tracking during the early stages of knee flexion, while the morphology of the trochlear groove and patellar face determines the relative position of the patella and femur as the knee flexion angle increases. Among the eight included studies that used musculoskeletal models to evaluate PFJF or PFJS, two musculoskeletal models were used. The first is the standard model available in simulation software (e.g., AMMR 1.6.2, MoCapModel) (Alexander and Schwameder, 2016), including patella, femur, and tendon, and the model was scaled to match each participant’s anthropometry and mass (Andersen et al., 2010). The second is a subject-specific musculoskeletal model based on subject-specific bone geometry, cartilage surface, and muscle fiber orientation (from MRI) (Chen and Powers, 2014; Lenhart et al., 2015a). Similarly, the included studies of PFJS evaluation using DEA and FEA also adopted the subject-specific model based on the radiation image. Using standardized models to evaluate PFJS may lack biofidelity. The subject-specific model may contribute to more accurately predicting patella tracking and PFJS evaluation (Mesfar and Shirazi-Adl, 2005).

During active knee flexion, the force of the quadriceps muscle is transmitted to the patellar tendon to generate stress on the contact surface of the patellofemoral joint. The researchers used several methods to estimate quadriceps muscle strength. The simplest method is to divide the knee extension moment by the effective lever arm of the quadriceps muscle (from radiography or magnetic resonance imaging). This method does not consider the co-activation of the hamstring and quadriceps during the activity. The second method used net joint moments and muscle moment arms (function of joint angles) to derive hamstring, quadriceps, and gastrocnemius muscle forces (Willson et al., 2015a; Willson et al., 2015b; Willy et al., 2016; Sinclair et al., 2018; Starbuck et al., 2021; Zavala et al., 2021). The third method is to modify the quadriceps muscle forces by the muscle’s cross-sectional area, maximum voluntary contraction force per unit cross-sectional area, and the EMG-force relationship (Escamilla et al., 2008a; Escamilla et al., 2008b; Escamilla et al., 2009). The fourth method is to estimate quadriceps muscle strength by summing the knee flexion moment and the net moment calculated by inverse kinematics. The knee flexion moment was estimated by SIMM software based on the individual’s lower extremity kinematics, the velocity of muscle contraction, and flexor muscle EMG (Chinkulprasert et al., 2011; Powers et al., 2014; Teng and Powers, 2014). The fifth method is computed muscle control algorithm that modifies muscle excitations by using feedforward and feedback control to follow recorded joint angle trajectories (Thelen et al., 2014). In addition, the researchers used static optimization techniques to estimate quadriceps muscle strength. Specifically, an active force adjustment, passive force adjustment, and velocity adjustment were interpolated from Arnold et al. (2010) curves for a given fiber length and velocity. The maximal dynamic muscle force was then calculated from the maximal isometric muscle force (Kernozek et al., 2015). From the perspective of the calculation principle, the quadriceps muscle force calculated by the coordinated contraction adjustment, computed muscle control algorithm, and static optimization techniques, while not flawless in their ability to represent muscle force *in vivo*, is more accurate than the force calculated from the net moment of knee extension and moment arm. However, it is still difficult to accurately solve the muscle force because the musculoskeletal system is redundant (the identical joint moment can frequently result from an unlimited number of combinations of muscle forces) (Johnson et al., 2022). And the motion simulation depends on experimental data and various uncertainty parameters, such as the variability of marker position, motion artifact, how to normalize EMG, and collecting EMG from deep muscles (Ackermann and van den Bogert, 2010; Burden, 2010). An earlier study has shown that the muscle is activated but does not produce effective muscle force in individuals with PFP (Herzog, 2000). Muscle force is a major factor affecting PFJS, and inaccurate quantification of muscle force may lead to misinterpretation of contact stress patterns. Islam et al. used three-dimensional registration techniques and linear mapping to measure the virtual penetration depth from the patellar cartilage surface to the femoral cartilage surface, combined with a FEA to estimate PFJS (Islam et al., 2015). The method provided by Islam et al. does not require the calculation of muscle force but provides accurate kinematics data of the patella and femur. However, the scanning space of MRI technology is narrow, and it is difficult to capture dynamic functional activities (walking, running, etc.). The development of biplane fluoroscopy systems has provided a method for directly measuring the kinematic data of the patella relative to the femur during functional activity (Wheatley et al., 2020). Gustafson et al. (2021) used a DEA model driven by high-precision kinematic data collected by a biplane fluoroscopy system to evaluate the PFJS during walk tasks. The method of DEA driven by high-precision kinematic data can estimate the PFJS at a subject-specific level without estimating the muscle force, avoiding the source of error. Therefore, the method for evaluating PFJS has a theoretically high accuracy. At the same time, this method can provide information on the change in the stress curve and distribution of the patellofemoral joint during the entire functional activity period, thereby promoting the understanding of PFP and the innovation of rehabilitation methods. The methods described are applicable for studying individuals with abnormal patellar trajectories and additional knee joint load.

FEA and DEA are refined computational models that allow the integration of subject-specific musculoskeletal parameters and experimental data, and are of great value for understanding stress distributions in complex biological structures (Fernandez et al., 2008; Farrokhi et al., 2011b; Gustafson et al., 2019). The researchers believe that shear stress is related to cartilage degeneration and damage, and higher shear stress in cartilage may also be the cause of damage to the exciting nociceptors present in the subchondral bone plate of the patella (Wojtys et al., 1990; (Besier et al., 2008). The FEA has been used to calculate the shear stress of PFP patients and healthy people during activities, and it was found that PFP patients have a higher level of shear stress (Farrokhi et al., 2011b). The studies of FEA and DEA included in this review conducted MRI or CT scans to obtain specific bone geometries to build subject-specific models. The material properties of cartilage are determined using constitutive models of varying complexity. These models range from linear spring models, which are described by a single constant, to isotropic, linear elastic models with two material constants, and a biphasic model with three material constants (Keenan et al., 2013). The studies used a linear elastic material model, which is widely accepted as valid due to the cartilage’s short-term elastic response during activities with loading frequencies over 0.1 Hz, such as walking (Higginson and Snaith, 1979). The average element size in the FEA was 0.5–1 mm, and previous studies on convergence analysis have shown that element sizes smaller than 0.75 mm did not result in significant stress changes but resulted in longer simulation times (Farrokhi et al., 2011b; Liao et al., 2015). The cartilage material properties of Young’s modulus and Poisson’s ratio are important factors affecting stress distribution of cartilage. Unfortunately, researchers do not seem to have reached a consensus about the material properties of cartilage. The minimum elastic modulus used in the included literature is 2 MPa, and the maximum is 40 MPa. Indentation testing is the main means of measuring the properties of materials. Although the indentation test is very accurate for determining material properties, researchers have not yet reached a consensus on cartilage properties. The thickness of cartilage, dehydration of the cartilage, contact area of the indentation test, and strain rate will affect the stiffness of cartilage. Kempson et al. reviewed the study of measuring the cartilage elastic modulus and gave the range of the cartilage elastic modulus under different working conditions (Kempson, 1980). Although the elastic modulus used in most studies related to PFJS evaluation is within this range, the elastic modulus used in some studies is beyond this range, and the basis for the elastic modulus was not provided. It should also be noted that changes in cartilage thickness will affect biomechanical properties (Shaktivesh et al., 2019). Individuals with PFP have been confirmed to have a possible loss of cartilage thickness (Farrokhi et al., 2011a). The rationality of using the same elastic modulus as that of healthy people to evaluate the PFJS of individuals with PFP needs further study. However, there is currently no research directly assessing the mechanical properties of articular cartilage in patients with PFP. The main technical limitation is the inability to directly estimate the mechanical performance of articular cartilage through non-invasive assessments such as MRI (Brenneman Wilson et al., 2023). Indirect prediction methods based on cartilage composition, such as quantitative MRI (Hatcher et al., 2017) and Raman spectroscopy (Bonifacio et al., 2010) and numerical simulations for performance prediction (Gupta et al., 2009), have shown promising potential in non-invasively assessing the mechanical properties of cartilage. In future research, these indirect prediction methods can be used to predict the mechanical properties of cartilage in patients with PFP, thereby promoting the development of stress calculation models for the patellofemoral joint.

In summary, the evaluation of PFJS using analytical models remains the most commonly used approach. Currently, researchers are enhancing the accuracy of PFJS evaluations by combining analytical models with MRI or musculoskeletal models. Musculoskeletal simulations, FEA, and DEA offer more detailed information on stress distribution. FEA can compute stress variations throughout the depth of the cartilage and estimate shear stress, which may be a key indicator of pain. Compared to analytical models, these three approaches consider more PFJS influencing factors and reduce sources of error. However, they are more time-consuming and require technical expertise. The evaluation of PFJS is undoubtedly a complex task. If the goal is to achieve high accuracy in evaluating PFJS, combining advanced imaging technologies (such as biplane fluoroscopy system) with advanced computational techniques (DEA and FEA) would be a good option. The method should be further developed to establish the “gold standard” for PFJS evaluation. If the focus is on exploring the impact of changes in muscle strength on PFJS, utilizing musculoskeletal models would be a suitable approach. For those seeking a simpler computational solution, analytical models would be appropriate. However, there is no clear boundary in the application scope of the aforementioned approaches. In practical applications, the appropriate technical means and evaluation approaches need to be selected based on theoretical considerations and/or ecological validity. Furthermore, the insufficient consideration of confounding factors remains a key limitation to the current research quality. There are multiple factors that impact PFJS such as sub-group classification of PFP patients (Selfe et al., 2013) and foot posture (Neal et al., 2014). In future studies, it is necessary to exercise better control over variables and minimize the influence of confounding factors. These measures will contribute to an improved level of evidence in research.

This study has potential limitations. A total of 69 studies were included in this review and the PFJF and PFJS data of included studies were extracted, but it was not possible to quantitatively analyze the calculation results between different methods due to the high heterogeneity (population, task).

## 5 Conclusion

There are four methods to evaluate patellofemoral joint reaction force and patellofemoral joint stress, including the analytical model, musculoskeletal model, discrete element analysis, and finite element analysis. At present, there is still no “gold standard” for PFJS. And researchers are still trying to improve the evaluation accuracy of PFJS. This is mainly achieved through using a personalized model and optimizing (or avoiding) quadriceps muscle strength calculation. In theory, the evaluation scheme of combining advanced computational and biplane fluoroscopy techniques has high accuracy in evaluating PFJS. Additionally, in practical applications, it is important to select the appropriate technical methods and evaluation approaches based on theoretical considerations and/or ecological validity. In the future, researchers can develop relevant model frameworks to accurately calculate PFJS and provide technical solutions for a better understanding of the mechanism of patellofemoral joint pain and optimization of the patellofemoral joint treatment program.

## Data Availability

The original contributions presented in the study are included in the article/Supplementary Material, further inquiries can be directed to the corresponding author.
