# Supplementary material for: The non-invasive evaluation technique of patellofemoral joint stress: a systematic literature review
Source: Front Bioeng Biotechnol. 2023 Jun 29;11:1197014. doi: 10.3389/fbioe.2023.1197014 (PMC10343958; doi:10.3389/fbioe.2023.1197014)
Supplement: Supplementary file 1 [file Table1.DOCX]

Supplementary Material

# Supplementary Figures and Tables

Supplementary table 1 Characteristics of the included studies

| Literature | | | Aim regarding PFJ stress | Participants Characteristics | | | | | | | | | | | | | | | | | | | | | | | | | | | | | | | |
| --- | --- | --- | --- | --- | --- | --- | --- | --- | --- | --- | --- | --- | --- | --- | --- | --- | --- | --- | --- | --- | --- | --- | --- | --- | --- | --- | --- | --- | --- | --- | --- | --- | --- | --- | --- |
|  |  |  |  | Participants | | | | | | | Sex | | | | | | | | | | | | | Age (yrs) | | | | | | | | High (cm) | | Weight (kg) | |
| Starbuck (2021) | | examined AT forces and PFJ stresses at different running speeds | | | | | | | | high-performing endurance runners | | | | F10/M10 | | | | | 25.1±7.6 | | | | | | | | 172.3±9.1 | | | | | | | 61.3±7.6 | |
| Zavala (2021) | | investigated differences in patellofemoral loading when experienced female lifters squatted to three depths and with three loads | | | | | | | | recreationally active females | | | | F19 | | | | | 25.1±5.8 | | | | | | | | - | | | | | | | 62.5±10.2 | |
| Gustafson (2021) | | aimed to evaluate the feasibility of this combined kinematic and DEA modeling framework for estimating unique patellofemoral stress profiles | | | | | | | | OA and healthy adults | | | | F2/M4 | | | | | 67.7±5.4 | | | | | | | | 177.8±13.0 | | | | | | | 74.0 ±9.4 | |
| Goulette (2021) | | examine PFJS comparing two movements: Forward Lunge (FL) and Backward Lunge (BL) | | | | | | | | healthy adults | | | | F20 | | | | | 22.6±1.9 | | | | | | | | 172.3±6.5 | | | | | | | 65.1±11.0 | |
| Kujawa (2020) | | analyze PFJS stress of running with and without a 9 kg load | | | | | | | | recreational runners | | | | F19 | | | | | 20.9±2.2 | | | | | | | | 171.7±6.7 | | | | | | | 64.9±9.6 | |
| Almonroeder (2020) | | evaluate the influence of verbal instructions regarding squat technique on patellofemoral joint loading | | | | | | | | recreationally active participants | | | | F11 | | | | | 22.7±5.5 | | | | | | | | 170±10 | | | | | | | 64.3±8.5 | |
| Thomeer (2020) | | describe and explain load distribution across the medial and lateral facets of the patella over one cycle of normal walking | | | | | | | healthy adults | | | | F3/M3 | | | | | 26.9±5.2 | | | | | | | | 171±10 | | | | | | | | 67.7±7.6 | |
| Wang (2020) | | | explore the differences in PFJS after 12-week gait retraining | recreational runners | | | | | | | M17 | | | | | | | | | | | | | CON: 27.6±5.2 EG: 32.4±6.1 | | | | | | | | CON:173.9±7.0 EG: 174.8±5.3 | | CON: 75.4±11.6 EG: 70.2±6.0 | |
| Atkins (2019) | | examined PFJS at different sagittal plane trunk posture during stair ascent | | | | | | | | asymptomatic females | | | | F20 | | | | | 23.4±2.5 | | | | | | | | 164.4±7.9 | | | | | | | 63.0±12.2 | |
| Dos Santos (2019) | | | calculate changes in PFJS during the different running conditions | recreational runners | | | | | | | F11/M8 | | | | | | | | | | | | | 28.05±5.03 | | | | | | | | - | | - | |
| Ristow (2019) | | | determine if there was a difference in PFJ loading variables between 2 hopping cadences (50 and 100 hops per minute) | healthy adults | | | | | | | F25 | | | | | | | | | | | | | 22.3±1.8 | | | | | | | | 171.4±6.3 | | 67.4±9.5 | |
| Liao (2019) | | | determine whether the location of peak patella cartilage stress differs between runners with and without PFP | recreational  runners | | | | | | | F22 | | | | | | | | | | | | | PFP: 27.6±5.3 CON: 27.4±5.2 | | | | | | | | PFP: 160±10 CON: 160±10 | | PFP: 54.6 ±6.0 CON: 58.4 ±6.3 | |
| Pal (2019) | | | Patellofemoral cartilage stresses are most sensitive to variations in vastus medialis muscle forces | PFP and healthy adults | | | | | | | F27/M10 | | | | | | | | | | | | | PFP  F: 27.4±4.0  M: 30.8±4.6 CON  F: 28.8±4.7  M: 27.8±2.8 | | | | | | | | PFP  F: 165±6  M: 178±9 CON  F: 166±5  M: 179±7 | | PFP  F: 58.9±9.2  M: 73.5±3.9 CON  F: 58.3±4.6  M: 73.2±4.2 | |
| Bonacci (2018) | | | determine the effect of a combination of a minimalist shoe and increased cadence on measures of PFJ | runners with PFP | | | | | | | F15 | | | | | | | | | | | | | 32.6±9.6 | | | | | | | | 171±6 | | 68.91±10.99 | |
| Boyer (2018) | | | compare joint loads between habitual RFS and habitual  FFS patterns, and shorter stride lengths | recreational or competitive runners | | | | | | | F8/M30 | | | | | | | | | | | | | RFS: 21±6 FFS: 22± 3 | | | | | | | | RFS: 182±8 FFS: 174±9 | | RFS: 72.0±10.7 FFS: 66.4± 10.3 | |
| Waiteman (2018) | | aimed at comparing PFJS of women with patellofemoral pain and pain-free controls during stair descent | | | | | | | | Females with patellofemoral pain and pain-free | | | | F64 | | | | | PFP: 21.6±2.9 CON: 21.8±3.0 | | | | | | | | PFP: 160±5 CON: 160±5 | | | | | | | PFP: 59.9 ±8.8 CON: 60.6 ±9.6 | |
| Sinclair (2018) | | explore the effects of a 4-week intervention using semi-custom insoles in 6 recreational runners with patellofemoral pain | | | | | | | | PFP | | | | F7/M10 | | | | | Strong: 33.64±6.8 Weak and tight: 34.83±12.59 | | | | | | | | Strong: 175±9 Weak and tight: 172±7 | | | | | | | Strong: 73.75±13.69 Weak and tight: 71.03± 13.71 | |
| Teng (2018) | | examine the location-specific correlations between peak PFJ stress during gait | | | | | | | | healthy population | | | | F17/M17 | | | | | 48.6 | | | | | | | | 24.9 kg/m^2^ | | | | | | | Teng (Teng et al., 2018) | |
| Ho (2018) | | compare PFJ stress between level, incline, and decline running | | | | | | | | recreational runners | | | | F10/M10 | | | | | 24.9±2.4 | | | | | | | | 170±7 | | | | | | | 67.0±9.7 | |
| Liao (2018a) | | | determine whether recreational runners with PFP exhibit greater peak PFJS compared to pain-free runners | | recreational runners | | | | | | | F22 | | | | | | | | | | | | | PFP: 27.6±5.3  CON: 27.4±5.5 | | | | | | | | PFP: 160±10 CON: 160±10 | | PFP: 54.6 ±6.0  CON: 58.8 ±6.5 |
| Liao (2018b) | | | determine the influence of femur and tibia rotations in the transverse and frontal planes on patella cartilage stress | | healthy adults | | | | | | | F6 | | | | | | | | | | | | |  | | | | | | | |  | |  |
| van Rossom (2018) | | | quantify knee loading during frequently used activities | | healthy adults | | | | | | | F7/M8 | | | | | | | | | | | | | 31±6 | | | | | | | | 22.35±1.54 kg/m^2^ | | |
| Hu (2018) | | | investigate the contact mechanics and kinematics of a natural knee joint during walking | a natural knee model | | | | | | | F1 | | | | | | | | | | | | | 70 | | | | | | | | 168 | | | |
| Kernozek (2018) | | | compare PFJS during squatting using two techniques: Squat while keeping the knees before the toes (SBT) and squat while allowing the knees to go past the toes (SPT) | | healthy adults | | | | | | | F25 | | | | | | | | | | | | | 23.69±0.74 | | | | | | | | 169.39±6.44 | | 61.55±9.74 |
| Esculier (2017) | | | explore the effect of the level of minimalist  shoes on PFJ of runners with PFP | runners with PFP | | | | | | | F43/M26 | | | | | | | | | | | | | 30.7±6.4 | | | | | | | | - | | - | |
| Almonroeder (2017) | | | compare the PFJ in males and females during running | recreationally runners | | | | | | | F18/M14 | | | | | | | | | | | | | F: 23.7±6.0  M: 25.0±5.6 | | | | | | | | F: 165±7 M: 182±5 | | F: 61.7 ±12.7 M: 81.6 ±12.6 | |
| Hofmann (2017) | explore the effect of the trunk and shank position on PFJ during the forward lunge | | | | | | recreationally active participants | | | | | | | | | F5/M13 | | | | | 34±13 | | | | | | | | 176.4±8.9 | | | | | 75.9±10.7 | |
| Alexander (2016) | analyses lower limb joint compression forces as well as tibiofemoral joint shear forces during sloped walking at different inclinations | | | | | healthy adults | | | | | | | | | M18 | | | | | 27.0±4.7 | | | | | | | | 180±5 | | | | | | 74.5±8.2 | |
| Sinclair (2016a) | | | examine the effects of minimalist, maximalist, and conventional footwear on the PFJ during running | runners | | | | | | | F20 | | | | | | | | | | | | | 24.24±3.21 | | | | | | | | 177±12 | | 78.20 ± 6.32 | |
| Sinclair (2016b) | | | investigate the effects of knee bracing on PFJ | | recreational athlete | | | | | | | F9/M11 | | | | | | | | | | | | | - | | | | | | | | - | | - |
| Willy (2016) | | | compare measures of PFJS across treadmill and overground running in healthy, uninjured runners. | | recreational runners | | | | | | | F9/M9 | | | | | | | | | | | | | 23.6±3.5 | | | | | | | | 22.2± 2.6 kg/m^2^ | | Willy (Willy et al., 2016) |
| Besier (2015) | | | determine whether patients with patellofemoral pain had elevated cartilage stress com-  pared with pain-free controls and test the hypothesis that females exhibit greater cartilage stress than males | PFP and healthy adults | | | | | | | PFP: F13/M11  CON: F8/M8 | | | | | | | | | | | | | PFP F: 29±5 M: 27±3 CON F: 29±5 M: 30±4 | | | | | | | | PFP F: 166±5 M: 179±7 CON F: 165±6 M: 178±9 | | PFP F: 58.3±4.6 M: 73.5±0.07 CON F: 60.4±9.1 M: 72.4±12.5 | |
| Kernozek (2015) | | | compare differences in PFJS from inverse dynamics (ID) and the combination of inverse dynamics and static optimization (IDSO) during squatting and running | | recreational runners | | | | | | | F11 | | | | | | | | | | | | | 22±1.8 | | | | | | | | 169±6.4 | | 64.2±4.9 |
| Vannatta (2015) | | | quantify differences in PFJS between RFS and FFS | | recreational runners | | | | | | | F17 | | | | | | | | | | | | | 22.8±3.17 | | | | | | | | 169±5.8 | | 63.7±5.4 |
| Peng (2015) | investigated the influence of PFP and fatigue on PFJ in female dancers | | | | | | ballet dancers | | | | | | | | | F25 | | | | | PFP: 18.3±0.5 CON: 18.2±0.4 | | | | | | | | PFP: 161.9±3.3 CON: 159.5±3.8 | | | | | PFP: 51.6±4.7 CON: 50.2±4.6 | |
| Liao (2015) | determine the influence of femur internal rotation on patella cartilage stress in females with PFP. | | | | | | PFP | | | | | | | | | F9 | | | | | 27.7±4.3 | | | | | | | |  | | | | | 63.3±8.4 | |
| Lenhart (2015a) | construct and validate a subject-specific knee model | | | | | | healthy adults | | | | | | | | | F1 | | | | | 23 | | | | | | | | 165 | | | | | 61 | |
| Lenhart (2015b) | used a computational model to investigate the influence of step rate manipulation on PFJS during running | | | | | | recreational  runners | | | | | | | | | F7/M15 | | | | | - | | | | | | | | 180±9 | | | | | 71.0 ±8.8 | |
| Islam (2015) | develop 3D FE models of the PF joint in order to quantify in vivo cartilage contact stress | | | | | | PFP and healthy adults | | | | | | | | | F12 | | | | | CON: 26± 4 PFP: 28±8 | | | | | | | | CON: 167.0±7.9 PFP: 167.0±4.7 | | | | | CON: 64.4 ± 5.7 PFP: 59.0 ± 5.5 | |
| Shah (2015) | determine the influence of hamstrings loading on  patellofemoral contact pressure | | | | | | five cadaveric knees | | | | | | | | | - | | | | | - | | | | | | | | - | | | | | - | |
| Sinclair (2015) | examined the influence of minimalist and conventional footwear on the PFJS | | | | | | adults | | | | | | | | | M10 | | | | | 22.38±4.47 | | | | | | | | 173±7 | | | | | 67.83 ± 5.65 | |
| Sinclair and Selfe, 2015 | | | determine whether female recreational runners exhibit distinct knee loading compared to males | recreational runners | | | | | | | F15/M15 | | | | | | | | | | | | | F: 27.67±7.52 M: 28.80±4.23 | | | | | | | | F: 167±19 M: 180±13 | | F: 63.33±10.07 M: 79.07±6.88 | |
| Willson (2015a) | | | examine sex differences in the effects of an exhaustive run on running mechanics | | recreational runners | | | | | | | F18/M17 | | | | | | | | | | | | | F: 22.9 M: 22.4 | | | | | | | | F: 168 M: 180 | | F: 59.7 M: 79.0 |
| Willson (2015b) | | | test for effects of foot strike pattern and step length on PFJ while running | | | | | healthy participants | | | | | | | | | F10/M10 | | | | | | F: 22.6 M: 22.6 | | | | | | | | F: 167 M: 181 | | | F: 57.5 M: 80.7 | |
| Chen (2014) | | | determine if persons with PFP exhibit differences in PJRF during functional activities | | | | healthy and PFP adults | | | | | | | | | F40 PFP:20 CON:20 | | | | | | CON: 26.1±7.2 PFP: 27.9±6.7 | | | | | | | | CON: 165.3±6.9 PFP: 168.1±5.8 | | | | CON: 59.1±7.2 PFP: 62.4±6.8 | |
| Lenhart (2014) | | | determine how altering step rate affects internal muscle forces and  patellofemoral joint loads | | | | recreational runners | | | | | | | | | F15/M15 | | | | | | 33±14 | | | | | | | | 175±11 | | | | 68.6±10.9 | |
| Sinclair (2014) | | | examined PFJ in barefoot and barefoot inspired footwear in relation to conventional running shoes | recreational runners | | | | | | | M30 | | | | | | | | | | | | | 26.21±5.52 | | | | | | | | 1.77±0.09 | | 73.54±6.00 | |
| Willson (2014) | | | examine the effects of changing step length during running on PFJ in females with and without patellofemoral pain. | recreational runners | | | | | | | F23 | | | | | | | | | | | | | CON: 21.0±2.3 PFP: 2 0.8±3.7 | | | | | | | | CON: 170±5 PFP: 169±5 | | CON: 61.2±6.0 PFP: 62.5±8.5 | |
| Bonacci (2014) | | | determine if running barefoot decreases PFJ in comparison to shod running | highly trained  runners | | | | | | | F8/M14 | | | | | | | | | | | | | 29.2±6.0 | | | | | | | | 176±7 | | 65.6±8.8 | |
| Powers (2014) | | | compare PFJ among weight-bearing and non–  weight-bearing quadriceps exercises | | healthy adults | | | | | | | F5/M5 | | | | | | | | | | | | | F: 25±1.0 M: 32.4±4.7 | | | | | | | | F: 167.8±4.3 M: 177.6±5.5 | | F: 56.5±4.5 M: 72.7±5.7 |
| Teng (2014) | | | examine the association between sagittal plane trunk posture and patellofemoral joint stress | | recreational runners | | | | | | | F12/M12 | | | | | | | | | | | | | F: 26.5± 6.4 M: 28.1± 7.2 | | | | | | | | F: 174±8 M: 174±8 | | F: 70.5 ± 7.0  M: 70.5 ± 7.0 |
| Elias (2013) | | | performed to evaluate the accuracy of computational assessment of the influence of the orientation of the patellar tendon on the patellofemoral pressure distribution | eight cadaveric knees | | | | | | | - | | | | | | | | | | | | | - | | | | | | | | - | | - | |
| Kulmala (2013) | | | examine whether runners using a forefoot strike pattern exhibit a different PFJ than runners who use rearfoot strike pattern | team sport athletes | | | | | | | F38 | | | | | | | | | | | | | FFS: 18.6±5.0 RFS: 17.5±3.6 | | | | | | | | FFS: 169±5 RFS: 169±5 | | FFS: 1.69±0.05 RFS: 62.8±8.6 | |
| Wirtz (2012) | | | compare PFJS during running among females with and without PFP | recreational runners | | | | | | | F38 | | | | | | | | | | | | | PFP: 21.3±2.6 CON: 21.6±4.4 | | | | | | | | PFP: 170±10 CON: 170±10 | | PFP: 62.9 ±7.7 CON: 61.8 ±9.2 | |
| Ho (2012) | | | determine if heel height increases patellofemoral joint loading during walking | healthy participants | | | | | | | F11 | | | | | | | | | | | | | 25.0± 3.1 | | | | | | | | 161.6± 5.4 | | 55.5±7.1 | |
| Chinkulprasert (2011) | | | quantify PFJS during forward step-up exercises | | healthy adults | | | | | | | F10/M10 | | | | | | | | | | | | | F: 24.1± 3.1 M: 25.8± 4.7 | | | | | | | | F: 160.7± 5.3 M: 176.1± 5.8 | | F: 54.4 ± 6.7 M: 69.4 ± 8.1 |
| Farrokhi (2011) | | | test the hypothesis that individuals with PFP exhibit greater patellofemoral joint stress profiles compared to persons who are pain-free | PFP and healthy adults | | | | | | | F20 | | | | | | | | | | | | | PFP: 27.7±4.3 CON: 27.0±4.4 | | | | | | | | PFP: 170±10 CON: 160±10 | | PFP: 63.3 ±8.4 CON: 61.9 ±8.7 | |
| Elias (2010) | | | evaluate a computational model used to characterize the influence of vastus medialis obliquus function on the patellofemoral pressure distribution | ten cadaveric knees | | | | | | | - | | | | | | | | | | | | | - | | | | | | | | - | | - | |
| Whyte (2010) | | | determine the effect of hamstring length on PFJS | recreationally active participants | | | | | | | M16 | | | | | | | | | | | | | 25.25±1.91 | | | | | | | | 176±6 | | 72.5±10.5 | |
| Escamilla (2009) | | | compare patellofemoral compressive force and stress during the one-leg squat and two variations of the wall squat | | healthy adults | | | | | | | F8/M8 | | | | | | | | | | | | | F: 25±2 M: 29±7 | | | | | | | | F: 164±6 M: 177±6 | | F: 60±4 M: 77±9 |
| Besier (2005) | | | presents a modeling pipeline to estimate in vivo cartilage stress in the PF joint. | healthy adults | | | | | | | 1 | | | | | | | | | | | | | U | | | | | | | | U | | U | |
| Escamilla (2008a) | | | compare patellofemoral compressive force and stress between forward and side lunges with and without a stride | | healthy adults | | | | | | | F8/M8 | | | | | | | | | | | | | F: 25±2 M: 29±7 | | | | | | | | F: 164±6 M: 177±6 | | F: 60±4 M: 77±9 |
| Escamilla (2008b) | | | compare PFJ between a short- and long-step forward lunge both with and without a stride | | healthy adults | | | | | | | F8/M8 | | | | | | | | | | | | | F: 25±2 M: 29±7 | | | | | | | | F: 164±6 M: 177±6 | | F: 60±4 M: 77±9 |
| Besier (2008) | | | determine the influence of femoral internal and external rotation on stresses in the patellofemoral cartilage | | healthy adults | | | | | | | F8/M8 | | | | | | | | | | | | | F: 29±5 M: 29±6 | | | | | | | | F: 165±5 M: 177±6 | | F: 574±5.1 M: 72.6±6.0 |
| Fernandez (2008) | | | describes the use of low-dose X-ray fluoroscopy, an in vivo dynamic imaging modality that is finding increasing application in human joint motion measurement | | healthy adults | | | | | | | M1 | | | | | | | | | | | | | 26 | | | | | | | | 180 | | 65 |
| Elias (2004) | | | characterize the accuracy of the model | | four cadaveric knees | | | | | | | - | | | | | | | | | | | | | - | | | | | | | | - | | - |
| Power (2004) | | | determine the influence of bracing on patellofemoral joint stress during stair ambulation | | PFP | | | | | | | F15 | | | | | | | | | | | | | 29.9±8.0 | | | | | | | | 163.8±4.6 | | 58.0±8.0 |
| Powers (2004) | | | test the hypothesis that individuals who respond favorably to  bracing would exhibit decreased patellofemoral joint  stress during level walking. | PFP | | | | | | | F15 | | | | | | | | | | | | | 29.9±8.0 | | | | | | | | 163.8±4.6 | | 58.0±8.0 | |
| Wallace (2002) | | | to quantify patellofemoral joint reaction forces and stress while squatting with and without an external load | healthy adults | | | | | | | F9/M6 | | | | | | | | | | | | | 26±5 | | | | | | | | 171±9 | | 72 ± 16 | |
| Brechter (2002b) | | | determine whether individuals PFP demonstrate elevated PFJS compared with pain-free controls during free and fast walking | PFP:10  healthy:10 | | | | | | | F10/M10 | | | | | | | | | | | | | PFP: 37.1±10.4  CON: 32±7.1 | | | | | | | | PFP: 167.9±17.8  CON: 167.2±4.4 | | PFP: 70.8 ±14.3  CON: 67.9 ±14.5 | |
| Brechter (2002a) | | | determine if persons with PFP demonstrate elevated PFJS during stair ascent and descent when compared to persons without PFP | PFP:10  healthy:10 | | | | | | | F10/M10 | | | | | | | | | | | | | PFP: 37.1±10.4  CON: 32±7.1 | | | | | | | | PFP: 167.9±17.8  CON: 167.2±4.4 | | | |
| Salem (2001) | | | to characterize the PFJS during squatting | collegiate women athletes | | | | | | | F5 | | | | | | | | | | | | | 19±1.4 | | | | | | | | 178±10.6 | | 73±10 | |

Note: PFP, patellofemoral pain; CON, control group; EG, experimental group; F, female; M, male; PFJ, patellofemoral joint; OA, osteoarthritis; U, unknow.

Supplementary table 2 Patellofemoral joint stress result by mathematical model

| Literature | Evaluated Activity | Knee flexion angle(°) | Knee extension moment (N•m•kg^-1^) | F_Q_ (BW) | PFJF (BW) | Contact area (mm^2^) | PFJS (Mpa) |
| --- | --- | --- | --- | --- | --- | --- | --- |
| Starbuck (2021) | ran overground at four running speeds (3.3, 3.9, 4.8, and 5.6 m/s) | 3.4 m/s: 41.73±4.75  3.9 m/s: 42.32±4.81  4.8 m/s: 41.89±5.67  5.6 m/s: 41.89±5.86 | 3.4 m/s: 1.52±0.22  3.9 m/s: 1.59±0.23  4.8 m/s: 1.62±0.30  5.6 m/s: 1.57±0.33 | - | 3.4 m/s: 6.98±1.47  3.9 m/s: 7.49±1.62  4.8 m/s: 7.77±1.92  5.6 m/s: 7.77±0.05 | - | 3.4 m/s: 8.35±1.11  3.9 m/s: 8.90±1.14  4.8 m/s: 9.27±1.49  5.6 m/s: 9.26±1.72 |
| Goulette (2021) | forward lunge. | 101.7±7.4 |  | 4.77±0.64 | 4.63±0.62 | - | 5.00±1.32 |
| Zavala (2021) | squat | - | 0.91±0.04 | - | 11.32±0.96 | - | 9.05±0.66 |
| Almonroeder (2020) | squat | - | - | - | 3.61±0.60 | - | 10.7±1.2 |
| Kujawa (2020) | run with at 3.46 m/s | 49.58±5.52 | 0.25±0.042 | 7.45±0.89 | 6.57±0.89 | - | 18.1±2.002 |
| Wang (2020) | run with 3.33 m/s | 35:4±2:9 | 1.96±0.54 | - | 4.43±1.59 | - | 12.08±4.02 |
| Atkins (2019) | ascended the stairs using their self-selected (SS), flexing (FLX), extending (EXT) trunk posture: | SS: 49.3±4.2  FLX: 46.6±4.0  EXT: 56.5±4.0 | SS: 1.1±0.3  FLX: 0.7±0.3  EXT: 1.4±0.2 | - | SS: 2.60±0.72 FLX: 1.57±0.65 EXT: 3.89±0.69 | SS: 237.1±2.6  FLX: 234.7±3.9  EXT: 240.1±0.9 | SS: 6.61±1.89  FLX: 4.02±1.79  EXT: 9.99±1.81 |
| Dos Santos (2019) | rearfoot strike (RFS), forefoot strike (FFS), 10% step rate increase (Step10%), and forward trunk lean (FTL) | RFS: 40.33±5.04 FFS: 37.48±5.81 Step10%: 37.50±4.03 FTL: 42.09±4.28 | RFS: 1.62±0.36 FFS: 1.19±0.47 Step10%: 1.55±0.25 FTL: 1.55±0.32 | - | - | - | RFS: 8.23±2.32 FFS: 6.00±2.71 Step10%: 7.26±2.16 FTL: 7.84±2.31 |
| Ristow (2019) | single-leg hopping | 50 HPM: 58.8±5.9  100 HPM: 57.9±5.4 | - | 50 HPM: 8.8±1.2  100 HPM: 9.2±1.4 | 50 HPM: 8.1±1.2  100 HPM: 8.6±1.4 | - |  |
| Teng (2018) | walk at a self-selected speed | - | - | - | - | - | PFJ OA: 3.1±2.7, 3.5  CON: 3.5±2.9, 4.1 |
| Bonacci (2018) | treadmill running  conditions: level, incline, and decline at a standardized speed of 2.3 m/s. | - | - | - | Incline: 4.10 ± 0.98  Level: 4.10 ± 0.98  Decline: 5.58± 1.14 | Incline: 234.8 ± 4.8 Level: 229.6 ± 7.8 Decline: 231.0 ± 6.5 | Incline: 11.6 ± 3.6 Level: 11.7 ± 3.4 Decline:15.9 ± 4.1 |
| Boyer (2018) | run with 3.35 m/s | - | - | - | RFS:10.8 ± 1.4 FFS:9.9 ± 2.0 | - | RFS:13.9 ± 2.6 FFS:12.0 ± 2.7 |
| Kernozek (2018) | squat | 92.9±8.3 | 0.10±0.01 | 4.2±0.52 | 2.4±0.4 | - | 3.4±0.5 |
| Waiteman (2018) | stair descent | - | - | - | PFP: 0.39±0.21  CON: 0.28±0.20 |  | PFP: 12.5±5.1 CON: 9.2±3.8 |
| Ho (2018) | run with 4 m/s | RFS:50.9±3.1 FFS:46.9±4.5 | RFS:3.54±0.69 FFS:3.13±0.77 |  | RFS:5.1±1.1 FFS:4.3±1.2 |  | RFS:13.0±2.8 FFS:11.1±2.9 |
| Sinclair (2018) | run with 4 m/s | Strong:38.86±4.44 Weak and tight: 41.54±10.59 |  |  | Strong:3.40±0.75  Weak and tight:3.68±1.30 |  | Strong:6.82±1.66 KPa/BW Weak and tight:7.66±2.64 KPa/BW |
| Esculier (2017) | control shoe at preferred cadence (SC); control shoe at +10% preferred cadence (IC); minimalist shoe at preferred cadence (SM); minimalist shoe at +10% preferred cadence (IM) | SC: 40.62±7.60 IC: 37.03±6.78 SM: 38.03±7.03 IM: 35.61±6.70 | SC: 1.23±0.29 IC: 1.08±0.19 SM: 1.08±0.22 IM: 0.95±0.95 | - | SC: 4.40±1.43 IC: 3.58±0.92 SM: 3.66±1.08 IM: 3.06±1.04 | - | SC: 12.27±2.92 IC: 12.27±2.92 SM: 10.39±1.93 IM: 10.39±1.93 |
| Almonroeder (2017) | running at preferred speed |  |  |  |  |  |  |
| Hofmann (2017) | forward forward lunge (FF); forward vertical lunge (FV); Vertical Vertical lunge (VV) | - | FF: 1.8 ± 0.4 FV: 1.5 ± 0.5 VV: 1.5 ± 0.3 | - | FF: 4.34±0.81 FV: 3.61±0.77 VV: 4.15±1.02 | - | FF: 7.17±1.52 FV: 6.38±1.52 VV: 7.34±1.52 |
| Willy (2016) | run at a self-selected speed | treadmill: 34.2±3.5 overground: 34.3±3.8 | treadmill: 1.18±0.20 overground: 1.14±0.27 |  | treadmill: 4.0 ± 1.0 overground: 4.0 ± 0.8 |  | treadmill: 6.2±1.4 overground: 6.1±1.5 |
| Sinclair (2016a) | run with 4 m/s | - | - | - | Female:  Brace: 2.98±0.78 No-brace: 3.82±0.56 Male: Brace: 3.21±0.93 No-brace: 3.40±o.68 | - | Female: Brace:9.41±2.00 No-brace:11.6±1.62 Male: Brace: 10.11±2.07 No-brace:10.87±2.74 |
| Sinclair (2016b) | run with 4 m/s | F: 45.6±4.5 M: 46.3±3.9 | F: 2.7±0.4 M: 3.1±0.4 | - | F: 5.95±1.24 M: 7.13±1.52 | - | F: 10.4±2.4 M: 15.8±3.4 |
| Kernozek (al., 2015) | squat | - | - | Squat trials ID:3.81±0.72 IDSO:5.16±0.82 Running trials ID:3.12±0.69 IDSO:10.10±1.03 | - | - | Squat trials ID:9.81±3.36 IDSO:17.06±4.34 Running trials ID:7.53±1.02 IDSO:15.18±1.41 |
| Willson (2015a) | run with 3.5 m/s | Before exhaustion F: 1.0±0.19 M: 0.95±0.22 After exhaustion F: 1.0±0.19 M: 0.97±0.23 |  |  | Before exhaustion F: 4.2±0.9 M: 4.1±1.0 After exhaustion F: 4.3±0.9 M: 4.2±0.8 |  | Before exhaustion F: 6.0±1.5 M: 5.7±1.3 After exhaustion F: 6.0±1.4 M: 5.9±1.3 |
| Willson (2015b) | run with 2.5-3.5 m/s | - | - | - | RFS: 4.0±0.7  FFS: 3.6±0.7 | - | RFS: 5.7±1.1  FFS: 5.1±1.0 |
| Peng (2015) | échappé | - | - | - | - | - | No fatigue  PFP: 7.22±1.03 CON: 6.53±1.00 Fatigue(Mpa/kg) PFP: 6.71±1.03  CON: 5.57±1.00 |
| Sinclair (2015) | landing from 40 cm box |  | minimalist: 3.94±0.94  conventional: 4.66±1.02 |  | minimalist: 8.56±1.68  conventional: 10.84±1.75 |  | minimalist: 20.83±3.25  conventional: 26.71±3.08 |
| Sinclair and Selfe, 2015 | run with 4 m/s | Maximalist:42.91±6.69 Conventional:42.76±5.12 Minimalist:39.98±6.95 | - | - | Maximalist:4.74±0.88 Conventional:4.70±0.91 Minimalist:3.87±1.0 | - | Maximalist:13.59±2.63 Conventional:13.34±2.43 Minimalist:11.59±2.63 |
| Vannatta (2015) | run with 3.52-3.89 m/s | FFS:50.98±5.02 RFS:51.6±4.77 | - | FFS:8.37±1.28 RFS:10.62±1.45 | - | - | FFS:12.39±1.85 RFS:15.76±2.45 |
| Powers (2014) | Squat (90°) | - | - | - | - | - | 12.3±1.6 |
| Teng (2014) | run at 3.4 m/s using 3 different trunk postures | Flexed:44.5±3.7 Self-selcted:43.6±3.5 Extend:46.5±4.6 | Flexed:3.29±0.34 Self-selcted:3.54±0.31 Extend:3.70±0.31 |  | Flexed:7.24±1.32  Self-selcted:7.65±1.04  Extend:8.30±1.30 | Flexed:232.5±4.2 Self-selcted:231.6±4.4 Extend:233.5±4.5 | Flexed:20.2±3.4  Self-selcted:21.5±3.2  Extend:23.1±3.4 |
| Sinclair (2014) | run with 4 m/s | - | F:3.47±0.25 M:3.04±0.30 | - | F:3.84±0.45 M:3.25±0.46 | - | F:9.27±1.36 M:7.96±1.30 |
| Willson (2014) | run with 4 m/s | - | Barefoot: 2.58±0.70  Inov-8: 2.69±0.75  Conventional: 3.15±0.73 | - | Barefoot: 3.19±1.04  Inov-8: 3.56±1.29  Conventional:4.11±1.19 | - | Barefoot: 9.24±3.37  Inov-8: 9.65±3.77  Conventional:10.28±3.33 |
| Bonacci (2014) | run at 3.7 m/s | healthy: 40.5±5.2  PFP: 39.4±5.0 | healthy: 1.48±0.17  PFP: 1.30±0.20 |  |  |  | healthy: 10.2±1.4  PFP: 9.3±2.3 |
| Kulmala (2013) | run with barefoot and cushion shoe at 4.47 m/s | barefoot: 48.5±3.4 shod: 50.7±3.8 | barefoot: 3.0±0.4 shod: 3.3±0.4 |  | barefoot: 67.5±10.9 shod: 76.4±12.2 | barefoot: 244.2±1.3 shod: 244.5±0.84 | barefoot: 18.2±4.3 shod: 20.6±4.8 |
| Ho (2012) | walk with three heel height (low: 1.27 cm, medium: 6.35 cm, and high: 9.53 cm) | low: 16.1 ±5.1  medium: 22.1 ±5.9  high: 26.4 ±6.1 | low: 0.4 ±0.2  medium: 0.4 ±0.2  high: 0.8 ±0.3 |  | low: 0.54±0.31  medium: 0.86±0.41  high: 1.30±0.61 | low: 157.0 ±19.1  medium: 180.0 ±20.6  high: 180.0 ±20.6 | low: 1.9±0.7 medium: 2.6±1.2 high: 3.6±1.5 |
| Wirtz (2012) | run between 3.52 and 3.89 m/s | PFP:43.9±5.0) Healthy:41.8±4.1) | PFP:1.30±0.2) Healthy: 1.27±0.2) |  |  |  | PFP:9.6±2.5) Healthy:9.6±2.5) |
| Chinkulprasert (2011) | ascending stairs | 37.09 |  |  |  |  | 9.49 |
| Whyte (2010) | squat down as far as comfortable and return to standing |  |  |  | 1.23±0.39 | 490.32±71.94 | 231.48±67.52 Pa/kg |
| Escamilla (2009) | one-leg squat |  |  |  | 4.32±1.07 |  | 7.09±1.65 |
| Escamilla (2008a) | forward lunge | - | - | - | - | - | 5.45±1.58 |
| Escamilla (2008b) | forward lunge long and forward lunge short | - | - | - | - | - | long step: 5.09±1.53  short step: 7.09±1.99 |
| Powers (2004) | self-selected speed walking |  |  |  | Non-Braced: 6.70 | Non-Braced: 395.05 | Non-Braced: 2.33 |
| Brechter (2002b) | self-selected fast walking (1.82 m/s) | PFP: 59.30±1.71 CON: 60.70±0.87 | PFP: 0.66±0.19 CON: 0.73±0.08 |  | PFP: 12.30±2.98 CON: 13.37±1.16 | PFP: 144.24±48.90 CON: 240.49±34.01 | PFP: 6.61±1.69 CON: 3.13±0.79 |
| Brechter (2002a) | ascending stairs |  | PFP: 0.69  CON: 1.05 |  | PFP: 1.76  CON: 3.62 |  | PFP: 6.46  CON: 6.97 |
| Wallace (2002) | squat (90°) |  | 0.59±0.17 |  | 2.43±0.62 |  | 8.88±2.07 |
| Salem (2001) | squat with load 22 kg |  | Shallow:1.75±0.43  Medium:1.77±0.43  Deep:1.91±0.52 |  |  |  | Shallow:10.9±3.07  Medium:12.34±3.01Deep:11.76±2.98 |
| Power (2004) | stair ascent | 66.97 | 1.04 | - | 25.53 N•Kg^-1^ | 385.1 | 4.24 |

Note: PFP, patellofemoral pain; CON, control group; ‘-’: not applicable; F, female; M, male

Supplementary table 3 Patellofemoral joint stress result by musculoskeletal model

| Literature | Evaluated Activity | PFJF （BW） | | | Contact area （mm^2^） | PFJS (MPa) |
| --- | --- | --- | --- | --- | --- | --- |
|  |  | Contact force | | Shear force |  |  |
| Thomeer (2020) | walking （1.33 m/s）, stair descent, stair ascent, and running （3.33 m/s）, | PFP  walking: 0.79±0.12  stair descent: 2.23±0.30  stair ascent:3.04±0.31  running: 4.5±0.51 | CON  walking: 1±0.13  stair descent: 2.90±0.33  stair ascent: 3.64±0.32  running: 5.59±0.54 | - | - | - |
| van Rossom (2018) | squats, lunges, walking stairs, and gait | squat: 3.07±1.27  gait: 1.02±0.33  ascent stair: 3.17±0.52  descent stair: 3.30±0.75  forward lung: 2.91±0.98 | | squat: 1.06±0.46  gait: 0.34±0.17  ascent stair: 1.01±0.19  descent stair: 1.18±0.28  forward lung: 1.06±0.34 | - | - |
| Hu (2018) | walk | 1.44 | | - | - | - |
| Alexander (2016) | walk at prefer speed | 0.57±0.3 | | - | - | - |
| Lenhart (2015a) | walk at speed of 1.1 m/s on a ramp at different inclination angles of 0°, ±6°, ±12° and ±18° | -18°: 3.96±1.05 -12°: 2.78±0.91 -6°: 1.46±0.66 0°: 0.86±0.36 6°: 1.48±0.54 12°: 2.88±0.84 18°: 4.24±1.04 | | - | - | - |
| Lenhart (2015b) | walk at prefer speed | - | | - | - | 2.8 |
| Chen (2014) | run at preferred speed  （2.83±0.52 m/s） | 4.44 | | - | 632.35 | 10.56 |
| Lenhart (2014) | run at preferred speed （2.81±0.38 m/s） | step rate 100% 5.87±1.03  110% step rate: 5.03±1.07  90% step rate: 6.72±1.16 | | - | - | - |

Note: PFP, patellofemoral pain; CON, control group; ‘-’: not applicable

Supplementary table 4 Patellofemoral joint stress result by discrete element analysis

| Literature | Evaluated Activity | PFJF （BW） | | Contact area （mm^2^） | PFJS (MPa) | |
| --- | --- | --- | --- | --- | --- | --- |
|  |  | Lateral facet | Medial facet |  | Lateral facet | Medial facet |
| Gustafson (2021) | gait condition （7 % grade, 0.75 m/s） | - | - | - | 6.81±0.45 | 3.36±2.00 |
| Elias (2013) | 40°, 60°, and 80°of knee flexion | - | - | 40°: 300±39.62  60°: 354.72±56.6  80°: 384.91±79.24 | 40°: 4.09±0.59  60°: 4.12±0.74  80°: 4.03±0.52 | 40°: 3.34±1.09  60°: 2.72±0.74  80°: 2.31±0.54 |
| Elias (2010) | 40°, 60°, and 80°of knee flexion | - | - | - | 40°: 3.70±0.60  60°: 3.00±0.68  80°: 2.55±0.84 | 40°: 3.02±0.95  60°: 2.87±0.65  80°: 2.42±0.67 |
| Elias (2004) | knee flexion | - | - | - | 3.6-4.4 MPa | |

Note: ‘-’: not applicable

Supplementary table 5 Patellofemoral joint stress result by finite element analysis

| Literature | Evaluated Activity | F_Q_ （BW） | PFJF （BW） | Contact area （mm^2^） | PFJS | | | |
| --- | --- | --- | --- | --- | --- | --- | --- | --- |
| Besier (2015) | stair ascent | PFP  F: 3.76±0.75  M: 3.34±1.05  CON  F: 3.77±0.73  M: 3.23±1.04 | - | - | Effective stress (J/m^3^) | | | |
|  |  |  |  |  | PFP  F: 2.38±1.43  M: 1.40±0.72 | | | CON  F: 2.11±0.82  M: 1.35±0.37 |
| Liao (2019) | stair ascent | - | - | - | Effective stress：6.16 MPa | | | |
| Pal (2019) | step-up task | - | - | - | 5~10 MPa | | | |
| Liao (2018b) | run with speed of 2.7m/s | - | - | - | Hydrostatic pressure (MPa) | | Shear Stress (MPa) | |
|  |  |  |  |  | PFP: 17.97±6.99  CON: 15.56±4.04 | | PFP: 12.31±6.78  CON: 12.15±4.78 | |
| Liao (2018a) | run with speed of 2.7m/s | - | PFP：6.59±1.01  CON：6.12±0.94 | PFP：287.1±63.5  CON：326.5±56.0 | Hydrostatic pressure (MPa) | | Shear Stress (MPa) | |
|  |  |  |  |  | PFP：21.2±5.6  CON：16.5±4.6 | | PFP：11.3±4.6  CON：8.7±2.3 | |
| Liao (2015) | squat position at 45° | - | - | - | 2.01±1.62 MPa | | | |
| Shah (2015) | squat position at 15° and 45° | - | - | - | Hydrostatic pressure (MPa) | | Shear Stress (MPa) | |
|  |  |  |  |  | 15°: 0.99±0.40  45°: 1.65±0.32 | | 15°: 0.42±0.11  45°: 0.72±0.09 | |
| Islam (2015) | 40°, 60°, and 80°of knee flexion | - | - | - | Lateral facet (MPa)  40°: 2.85±0.57  60°: 2.89±0.74  80°: 3.11±0.85 | | Medial facet (MPa)  40°: 2.42±0.46  60°: 1.99±0.45  80°: 1.77±0.37 | |
| Farrokhi (2011) | 45°of knee flexion | - | - | - | Hydrostatic pressure (MPa) | | Shear Stress (MPa) | |
|  |  |  |  |  | PFP: 3.2±0.8  CON: 2.7±0.7 | | PFP: 1.3±0.7  CON: 0.9±0.3 | |
| Besier (2008) | squat position （10 s） at 45° of knee flexion | - | - | - | Hydrostatic pressure (MPa) | | Shear Stress (MPa) | |
|  |  |  |  |  | PFP: 2.0±0.5  CON: 2.7±0.7 | | PFP: 1.3±0.7  CON: 0.9±0.3 | |
| Fernandez (2008) | squat position at 40° | - | - | - | 4.10 MPa | | | |
| Besier (2005) | squat position at 60° | - | - | - | Hydrostatic pressure (MPa) | Shear Stress (MPa) | | |
|  |  |  |  |  | 2.31 MPa | 1.16 MPa | | |

Note: PFP, patellofemoral pain; CON, control group; ‘-’: not applicable; F, female; M, male

Supplementary table 6 PRISMA checklist

| **Section and Topic** | **Item #** | **Checklist item** | **Location where item is reported** |
| --- | --- | --- | --- |
| **TITLE** | | |  |
| Title | 1 | Identify the report as a systematic review. | Page 1 |
| **ABSTRACT** | | |  |
| Abstract | 2 | See the PRISMA 2020 for Abstracts checklist. | Lines 8-27 |
| **INTRODUCTION** | | |  |
| Rationale | 3 | Describe the rationale for the review in the context of existing knowledge. | Lines 29-59 |
| Objectives | 4 | Provide an explicit statement of the objective(s) or question(s) the review addresses. | Lines 60-65 |
| **METHODS** | | |  |
| Eligibility criteria | 5 | Specify the inclusion and exclusion criteria for the review and how studies were grouped for the syntheses. | Lines 77-81 |
| Information sources | 6 | Specify all databases, registers, websites, organisations, reference lists and other sources searched or consulted to identify studies. Specify the date when each source was last searched or consulted. | Lines 70-75 |
| Search strategy | 7 | Present the full search strategies for all databases, registers and websites, including any filters and limits used. | Table 1 |
| Selection process | 8 | Specify the methods used to decide whether a study met the inclusion criteria of the review, including how many reviewers screened each record and each report retrieved, whether they worked independently, and if applicable, details of automation tools used in the process. | Lines 82-88 |
| Data collection process | 9 | Specify the methods used to collect data from reports, including how many reviewers collected data from each report, whether they worked independently, any processes for obtaining or confirming data from study investigators, and if applicable, details of automation tools used in the process. | Lines 99-106 |
| Data items | 10a | List and define all outcomes for which data were sought. Specify whether all results that were compatible with each outcome domain in each study were sought (e.g. for all measures, time points, analyses), and if not, the methods used to decide which results to collect. | Lines 99-102 |
|  | 10b | List and define all other variables for which data were sought (e.g. participant and intervention characteristics, funding sources). Describe any assumptions made about any missing or unclear information. | Supplementary tables 2-5 |
| Study risk of bias assessment | 11 | Specify the methods used to assess risk of bias in the included studies, including details of the tool(s) used, how many reviewers assessed each study and whether they worked independently, and if applicable, details of automation tools used in the process. | Lines 90-97 |
| Effect measures | 12 | Specify for each outcome the effect measure(s) (e.g. risk ratio, mean difference) used in the synthesis or presentation of results. | Supplementary tables 2-5 |
| Synthesis methods | 13a | Describe the processes used to decide which studies were eligible for each synthesis (e.g. tabulating the study intervention characteristics and comparing against the planned groups for each synthesis (item #5)). | N/A |
|  | 13b | Describe any methods required to prepare the data for presentation or synthesis, such as handling of missing summary statistics, or data conversions. | N/A |
|  | 13c | Describe any methods used to tabulate or visually display results of individual studies and syntheses. | N/A |
|  | 13d | Describe any methods used to synthesize results and provide a rationale for the choice(s). If meta-analysis was performed, describe the model(s), method(s) to identify the presence and extent of statistical heterogeneity, and software package(s) used. | N/A |
|  | 13e | Describe any methods used to explore possible causes of heterogeneity among study results (e.g. subgroup analysis, meta-regression). | N/A |
|  | 13f | Describe any sensitivity analyses conducted to assess robustness of the synthesized results. | N/A |
| Reporting bias assessment | 14 | Describe any methods used to assess risk of bias due to missing results in a synthesis (arising from reporting biases). | Lines 90-97 |
| Certainty assessment | 15 | Describe any methods used to assess certainty (or confidence) in the body of evidence for an outcome. | Lines 93-95 |
| **RESULTS** | | |  |
| Study selection | 16a | Describe the results of the search and selection process, from the number of records identified in the search to the number of studies included in the review, ideally using a flow diagram. | Figure 1 |
|  | 16b | Cite studies that might appear to meet the inclusion criteria, but which were excluded, and explain why they were excluded. | No report |
| Study characteristics | 17 | Cite each included study and present its characteristics. | Tables 5-8 |
| Risk of bias in studies | 18 | Present assessments of risk of bias for each included study. | Tables 2-4 |
| Results of individual studies | 19 | For all outcomes, present, for each study: (a) summary statistics for each group (where appropriate) and (b) an effect estimate and its precision (e.g. confidence/credible interval), ideally using structured tables or plots. | Lines 122-272, Supplementary tables 2-5 |
| Results of syntheses | 20a | For each synthesis, briefly summarise the characteristics and risk of bias among contributing studies. | Lines 118-122 |
|  | 20b | Present results of all statistical syntheses conducted. If meta-analysis was done, present for each the summary estimate and its precision (e.g. confidence/credible interval) and measures of statistical heterogeneity. If comparing groups, describe the direction of the effect. | Supplementary tables 2-5 |
|  | 20c | Present results of all investigations of possible causes of heterogeneity among study results. | N/A |
|  | 20d | Present results of all sensitivity analyses conducted to assess the robustness of the synthesized results. | N/A |
| Reporting biases | 21 | Present assessments of risk of bias due to missing results (arising from reporting biases) for each synthesis assessed. | Tables 2-4 |
| Certainty of evidence | 22 | Present assessments of certainty (or confidence) in the body of evidence for each outcome assessed. | N/A |
| **DISCUSSION** | | |  |
| Discussion | 23a | Provide a general interpretation of the results in the context of other evidence. | Lines 274-426 |
|  | 23b | Discuss any limitations of the evidence included in the review. | Lines 426-437 |
|  | 23c | Discuss any limitations of the review processes used. | Lines 462-465 |
|  | 23d | Discuss implications of the results for practice, policy, and future research. | Lines 438-461 |
| **OTHER INFORMATION** | | |  |
| Registration and protocol | 24a | Provide registration information for the review, including register name and registration number, or state that the review was not registered. | Unregistered |
|  | 24b | Indicate where the review protocol can be accessed, or state that a protocol was not prepared. | Not prepared |
|  | 24c | Describe and explain any amendments to information provided at registration or in the protocol. | N/A |
| Support | 25 | Describe sources of financial or non-financial support for the review, and the role of the funders or sponsors in the review. | Lines 489-491 |
| Competing interests | 26 | Declare any competing interests of review authors. | Lines 485-491 |
| Availability of data, code and other materials | 27 | Report which of the following are publicly available and where they can be found: template data collection forms; data extracted from included studies; data used for all analyses; analytic code; any other materials used in the review. | No reported |
